# Design, Synthesis, and In Silico Multitarget Pharmacological Simulations of Acid Bioisosteres with a Validated In Vivo Antihyperglycemic Effect

**DOI:** 10.3390/molecules26040799

**Published:** 2021-02-04

**Authors:** Elix Alberto Domínguez-Mendoza, Yelzyn Galván-Ciprés, Josué Martínez-Miranda, Cristian Miranda-González, Blanca Colín-Lozano, Emanuel Hernández-Núñez, Gloria I. Hernández-Bolio, Oscar Palomino-Hernández, Gabriel Navarrete-Vazquez

**Affiliations:** 1 Facultad de Farmacia, Universidad Autónoma del Estado de Morelos, Cuernavaca, Morelos 62209, Mexico; ElixRose@outlook.com (E.A.D.-M.); yelzyn.galvanc@uaem.edu.mx (Y.G.-C.); josue.martinezm@uaem.edu.mx (J.M.-M.); cristian.mirandag@uaem.edu.mx (C.M.-G.); clbi_ff@uaem.mx (B.C.-L.); 2 Cátedra CONACyT, Departamento de Recursos del Mar, Centro de Investigación y de Estudios Avanzados, IPN, Unidad Mérida, Yucatan 97310, Mexico; emanuel.hernandez@cinvestav.mx (E.H.-N.); hboliog@gmail.com (G.I.H.-B.); 3 Computational Biomedicine (IAS-5/INM-9), Forschungszentrum Juelich, 52425 Julich, Germany; o.palomino@fz-juelich.de; 4 Department of Chemistry, Rheinisch-Westfälische Technische Hochschule Aachen, 52425 Aachen, Germany

**Keywords:** multitarget ligands, drug design, diabetes, molecular dynamics

## Abstract

Substituted phenylacetic (**1–3**), phenylpropanoic (**4–6**), and benzylidenethiazolidine-2,4-dione (**7–9**) derivatives were designed according to a multitarget unified pharmacophore pattern that has shown robust antidiabetic activity. This bioactivity is due to the simultaneous polypharmacological stimulation of receptors PPARα, PPARγ, and GPR40 and the enzyme inhibition of aldose reductase (AR) and protein tyrosine phosphatase 1B (PTP-1B). The nine compounds share the same four pharmacophore elements: an acid moiety, an aromatic ring, a bulky hydrophobic group, and a flexible linker between the latter two elements. Addition and substitution reactions were performed to obtain molecules at moderated yields. In silico pharmacological consensus analysis (PHACA) was conducted to determine their possible modes of action, protein affinities, toxicological activities, and drug-like properties. The results were combined with in vivo assays to evaluate the ability of these compounds to decrease glucose levels in diabetic mice at a 100 mg/kg single dose. Compounds **6** (a phenylpropanoic acid derivative) and **9** (a benzylidenethiazolidine-2,4-dione derivative) ameliorated the hyperglycemic peak in a statically significant manner in a mouse model of type 2 diabetes. Finally, molecular dynamics simulations were executed on the top performing compounds to shed light on their mechanism of action. The simulations showed the flexible nature of the binding pocket of AR, and showed that both compounds remained bound during the simulation time, although not sharing the same binding mode. In conclusion, we designed nine acid bioisosteres with robust in vivo antihyperglycemic activity that were predicted to have favorable pharmacokinetic and toxicological profiles. Together, these findings provide evidence that supports the molecular design we employed, where the unified pharmacophores possess a strong antidiabetic action due to their multitarget activation.

## 1. Introduction

**Figure 1:**
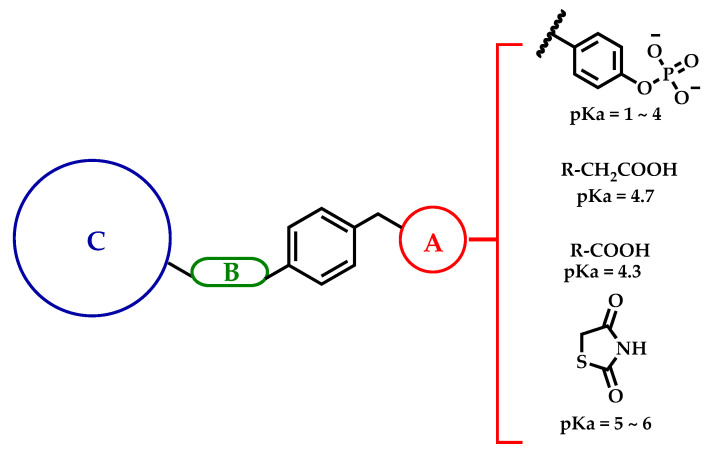
Unified antidiabetic pharmacophore proposed with multitarget activity on PPARα, PPARγ, GPR40, aldose reductase (AR), and PTP-1B: (A) acid group, (B) flexible linker, (C) bulky hydrophobic group, and a central phenyl core. A pKa comparison between acid bioisosteric groups used to design these molecules is also shown.

Hyperglycemia and insulin resistance are hallmarks of diabetes; the prevalence of diabetes is almost 425 million people globally, but this number would be twice that if it considered the people who are currently undiagnosed [1]. Inactivity, a hypercaloric diet, and lack of exercise have aggravated the diabetes epidemic, and this disease has a large impact on the quality of life of the people who are diagnosed with it [2]. The current pharmacotherapy to treat diabetes consists of a wide roster of drugs that have a variety of modes of action [3]. Secretagogues, insulin sensitizers, glucose-uptake improvers, glucose-reuptake blockers, and glucose absorption inhibitors are the main drug families that are currently sold as pharmacological therapies [4]. However, the use of these drugs has been limited due to their side effects and the fact that they have not been able to control blood glucose in the diabetic population. The failure of these drugs to maintain blood glucose at healthy levels has led to research on new drugs with novel modes of action. GPR-40 (G-protein-coupled receptor 40), PTP-1B (protein tyrosine phosphatase-1B), and aldose reductase (AR) are the targets that have received increased attention in recent years [5]. GPR-40 agonists have a secretagogue effect to increase glucose-dependent insulin secretion. PTP-1B belongs to a family of proteins that catalyze the hydrolysis of phosphorylated tyrosine residues and can therefore modulate insulin signaling, which decreases insulin resistance [6]. AR reduces glucose to sorbitol, but excessive levels of this molecule enhance osmotic pressure and cellular stress [7], which can lead to diabetic complications such as nephropathies, neuropathies, and cardiomyopathies. AR inhibitors have been proposed as a means to ameliorate these diabetic complications [5,7]. Currently, none have received market approval in territories outside of India and China [8]. From our medchem lab, a unified pharmacophore has been proposed (Figure 1) and has shown robust multitarget antidiabetic action over PPARα, PPARγ, GPR40, AR, and PTP1B, five proteins implicated in diabetes [5,9,10]. This multitarget unified pharmacophore is integrated by an acid moiety, an aromatic ring, a flexible linker group, and a bulky hydrophobic group [11]. Previous work by our research group and others has shown that molecules with these pharmacophore characteristics can interact with high affinity with several proteins of interest for the treatment of diabetes. In particular, they display a simultaneous multitarget stimulation of receptors PPARα, PPARγ, and GPR40 and the enzyme inhibition of AR and PTP1B [5,9,10,11,12,13]. Thus, the preparation of nine acid bioisosteres (chemical substituents with similar physicochemical properties that produce broadly similar biological properties related to another chemical entity), the in vivo antihyperglycemic activity of these compounds in streptozotocin-induced diabetic mice, and the molecular docking and dynamics prediction of this mode of binding over PTP-1B and AR enzymes are reported in this work. Altogether, this study supports the potential of these nine acid bioisosteres (derived from phenylacetic and phenylpropanoic acids as well as benzylidenethiazolidine-2,4-dione) to control blood glucose through a “multitarget activity” approach for the experimental treatment of diabetes.

## 2. Results and Discussion

### 2.1. Drug Design

Figure 1 shows the unified multitarget pharmacophore designed in our lab, which has shown robust antidiabetic activity in previous research [5,9,10,11,12,13]. We suggest that the four colored moieties are needed to exert the aforementioned multitarget effect on PPARα, PPARγ, GPR40, AR, and PTP-1B [5,10,13]. These groups participate in the interaction between the molecules and the binding pocket of the protein. The acid head group was considered due to its phosphoric acid-like characteristics; the linker with the free rotation allows an adequate arrangement in the binding pocket of the protein, and the bulky hydrophobic group increases the contact surface area to form a larger number of interactions within the pocket [11].

### 2.2. Chemistry

**Figure 2:**
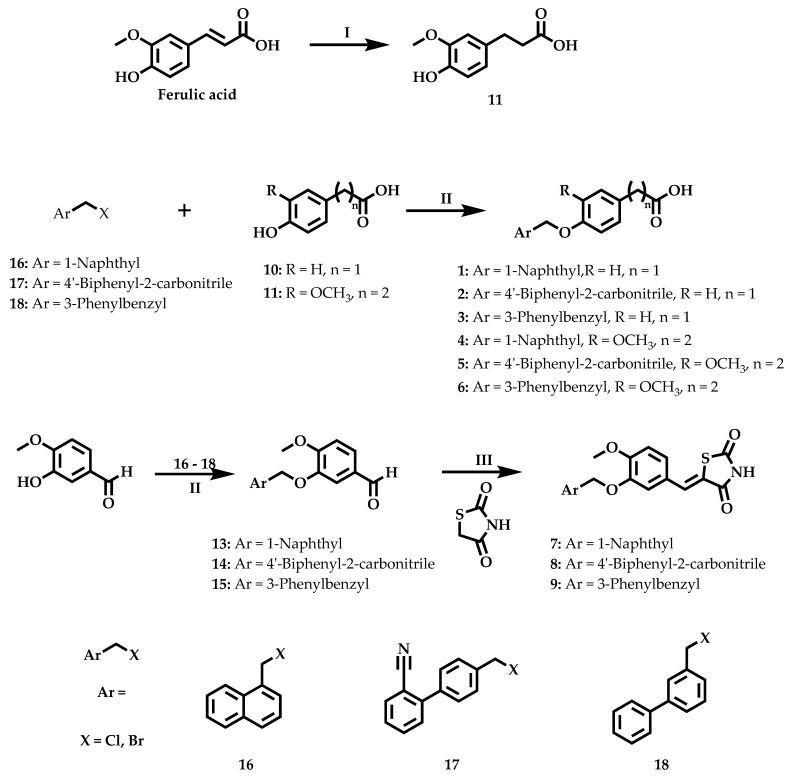
Synthetic route to achieve the desired compounds: (**I**) H_2_, Pd^0^/C, EtOH, R.T.; (**II**) K_2_CO_3_, CH_3_CN, reflux; (**III**) Benzoic acid (0.3 eq.), piperidine (0.3 eq.), toluene, Dean–Stark apparatus, reflux.

The synthesis of the compounds was conducted according to Figure 2. We used three different reagents to start the synthesis: ferulic acid, (4-hydroxyphenyl)acetic acid (**10**), and isovanillin (**12**). In the case of the ferulic acid, we decided to perform a reduction of the double bond using catalytic hydrogenation to improve the free rotation of the final compound and to decrease the reactivity and the intrinsic toxicity of the α,β—unsaturated carbonyl, obtaining Compound **11** (Figure 2, Step I). After, we performed a bimolecular nucleophilic substitution to join the region with the bulky hydrophobic group of arylmethylhalides with phenolic acids or aldehyde (Figure 2, Step II). Lastly, to generate another acid bioisosteric group, a Knoevenagel condensation was performed between isovanillin (**12**) and thiazolidine-2,4-dione using a Dean–Stark apparatus to remove the water formed in the reaction (Figure 2, Step III).

### 2.3. In Silico Analysis

#### 2.3.1. Structural Analysis of the Targets

Molecular dynamics simulations are used to understand biomolecular structure and dynamics [14,15,16]. In particular, for protein–ligand interactions, they have shed light on multiple venues, ranging from the structure–activity relationships of multiple compounds [17] to the kinetics and thermodynamics of binding [18].

**Figure 3:**
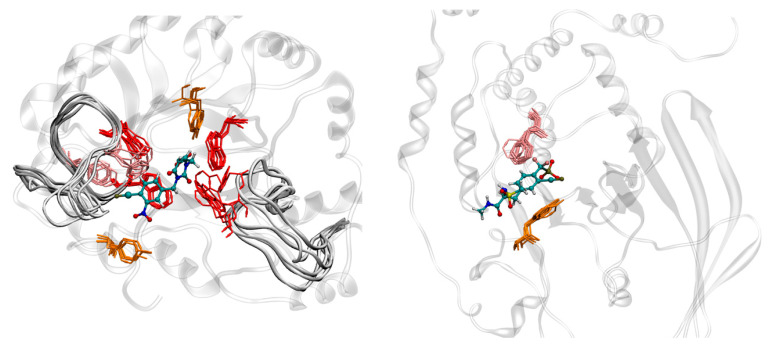
Snapshots of the Molecular Dynamics (MD) simulations of AR (**left**) and PTP-1B (**right**) with their respective cocrystallized ligands. The protein structure is shown in the white transparent illustration, ligands in CPK representation, and aromatic residues close to the binding pocket in licorice representation (red: tryptophan; orange: tyrosine; pink: phenylalanine). The superimposed snapshots illustrate the plasticity of the loops (and its aromatic residues) close to the binding pocket of AR.

MD simulations of the enzymes AR and PTP-1B were performed in the *holo* state (see Materials and Methods for details). Figure 3 shows the binding pockets of the selected molecular targets during the MD simulation, with snapshots every 50 ns. For AR, the simulation showed high flexibility for the loops composed of Residues 112–136 and 213–228. Given the closeness to the binding site, this suggests that these loops are adaptable to larger ligands. This is further supported by the presence of aromatic residues in said loops, which could support binding via hydrophobic interactions. The binding pocket shows two anchors for negative charges through one side with the backbone of Leu300 and the other with the side chains of Tyr48, His110, and Lys77. Additional π-stacking interactions are provided by Trp111, in line with a previous work [19]. In the case of PTP-1B, the receptor shows a more rigid topology, with less aromatic/hydrophobic residues, and a smaller binding pocket. This pocket consists of an anchor for negative charges composed of Ser216, Ala217, Gly218, Ile219, Gly220, and Arg221 and additional interactions through π-stacking with Phe182.

#### 2.3.2. Pharmacodynamics Predictions

Molecular docking was performed with the enzymes PTP-1B and AR. In order to incorporate the flexibility of the receptor into the analysis, an ensemble docking approach was performed using the six most representative structures of the MD trajectories. All the compounds tested were shown to interact with both receptors, although with putative different affinity, shown both in terms of docking score and ligand efficiency. We considered the bioisosteric groups to be deprotonated at pH 7.4.

The interactions that we found between the synthesized compounds and the two proteins are summarized in Table S1 (Supplemental Information). In the case of AR, Compounds **1** and **4–9** showed interactions similar to the cocrystallized ligand, such as those established with Tyr48, Leu300, and Trp111. The latter contact has been reported as one of the main binding residues in previous AR inhibitors [5]. Moreover, the compounds studied here form more interactions with the aromatic residues of the loop regions, such as Trp20, Phe122, and Trp219. For PTP-1B, most compounds interact similarly with the residues involved in the catalytic triad constituted by Arg221, Asp181, and Cys215 residues [12,13]. These residues are key for substrate binding, improving complex stability, and performing the nucleophilic attack [20]. All the reported compounds interact with the aforementioned negative charge anchor (Residues 216–221) through their negative moiety, and leave the biphenyl group solvent-exposed. In general, the majority of compounds presented satisfactory binding scores and interactions, which made them amenable for further studies.

#### 2.3.3. Pharmacokinetics and Toxicological Properties

With the purpose of anticipating potential off-targets, adverse effects and toxicity are associated with Compounds **1–9**, a virtual prediction of their safety profiles was calculated using the web servers AdmetSAR [21], SwissADME [22], and ACD/ToxSuite software, v. 2.95 (Tables S2 and S3). We obtained the values of the different properties using in silico tools to determine whether these molecules were appropriate for animal model testing. From all the tested molecules (Table S2), the thiazolidine-2,4-diones **7–9** showed the best intestinal absorption values. However, their toxicological profiles indicate that these molecules could be hERG blockers and thus show certain degrees of cardiotoxicity, in line with other examples in the literature on the toxicity of the thiazolidine-2,4-dione moiety [23,24,25]. Despite all that, research on this structural motif continues due to the beneficial effects of these drugs, including preservation of the pancreas’s ability to produce sufficient levels of insulin and improve lipid metabolism by increasing levels of HDL [26,27]. Moreover, rosiglitazone, which is a thiazolidine-2,4-dione previously withdrawn from the market, returned to it last year [28]. In the calculation of acute toxicity (Table S3), Compounds **1–9** demonstrated similar or even higher predicted LD_50_ values than the antidiabetic drugs pioglitazone (PIO) and glibenclamide (GLI), regardless of the administration route in mice and rats: oral (p.o.) or intraperitoneal (i.p.). Therefore, they were predicted to be less toxic than the common antidiabetic drugs that were used as a reference in this work (Table S3). The predicted inhibition of the three main cytochrome P450 isoforms for Compounds **1–9** were similar to those of pioglitazone and glibenclamide at relevant clinical concentrations (less than 10 μM), indicating low probabilities of drug–drug interactions. Overall, the pharmacodynamics and pharmacokinetic predictions suggested that the synthesized compounds possessed acceptable properties for further in vivo studies.

### 2.4. Antidiabetic Assay

After the synthesis and in silico analysis, we conducted the in vivo assay. We used a streptozotocin (STZ)-nicotinamide (NA)-induced diabetic mouse model to determine which compounds had an antidiabetic activity (Table 1). Compounds **2**, **6**, **8**, and **9** exerted a clearly antihyperglycemic effect since they prevented the hyperglycemic peak at the first hour in a statistically significant manner in contrast to the control group.

**Figure 4:**
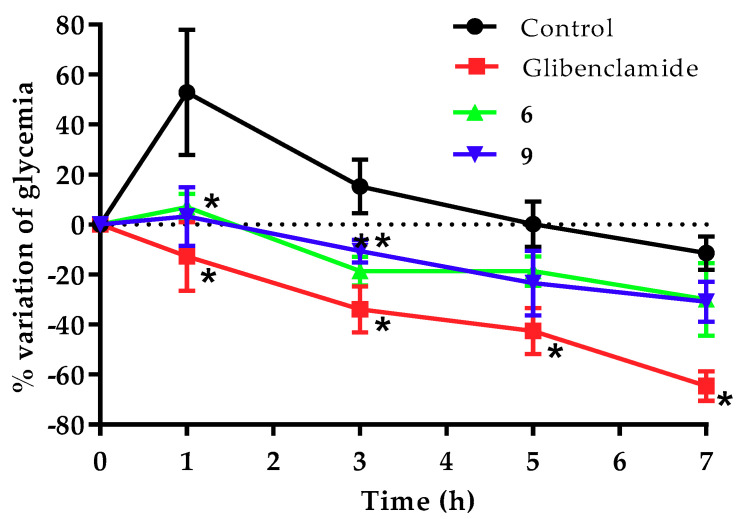
Effect of a single dose of **6** and **9** over the blood glucose in STZ-NA-induced diabetic mice. * Statistically significant difference versus control group by multiple comparison Dunnett’s test. Glibenclamide was administered at 20 mg/kg. Compounds were administered at 100 mg/kg (*n* = 6, mean ± SEM, *p* < 0.05).

Phenol and acid natural products isolated from marine sponge and plants have been described in the literature to decrease blood glucose by the inhibition of PTP-1B [29,30]. We focused on the design and synthesis of these acid molecules with antihyperglycemic activity. Figure 4 shows that Compounds **6** and **9** prevented the increase in glucose concentration since the first hour post-administration, which was maintained throughout the rest of the experiment, even though their effects are not as strong as the positive control. Compounds **4**, **5**, and **7** only prevented the increase in glycemia and did not have additional effects beyond maintaining the blood glucose in a range that was close to baseline values; this effect is known to be one of the major regulators of energy in vivo [31]. In contrast, the positive control glibenclamide reduced the blood glucose levels in a significant manner after 7 h of the assay (−64.6%), unlike Compounds **6** and **9** (−30.0% and −30.9%, respectively). These findings are positive because, unlike the secretagogue drugs, these compounds will not result in hypoglycemia.

Interestingly, the two compounds with the best activity (**6** and **9**) share the same bulky group (3-phenylbenzyl motif). Compound **6** contains a propanoic acid that is attached to the central aromatic ring, whereas Compound **9** possesses a double bond at position five of the thiazolidine-2,4-dione ring, which results in a conformationally stable molecule because the double bond is restricted in its rotation [32]. This is consistent with previous reports [5], where phenylpropanoic acids with bulky and lipophilic groups showed an antidiabetic effect but were mediated by GPR-40 and PPAR activation. In contrast, when an electron-withdrawing substituent on the bulky group such as cyano was present in Compounds **2**, **5**, and **8**, the in vivo biological activity was reduced. On the other hand, cutting the chain from three carbon atoms (phenylpropionic) to two (phenylacetic) in the acidic region caused a decrease in antidiabetic activity for Compounds **1–3**.

### 2.5. Pharmacological Consensus Analysis

We performed an in silico pharmacological consensus analysis (PHACA). The data are reported in Table 2 using a traffic light system. PHACA combines the results of the previous pharmacodynamics and pharmacokinetic predictions, toxicity predictions, and additional experimental data. The rationale for a pharmacological consensus analysis is that, when more predicted parameters agree that a compound is active and has low toxicity and an adequate pharmacokinetic profile, the selection of a compound with suitable pharmacological behavior for synthesis is more reliable. Therefore, a compound that has a high score from a collection of multiple predictions is more likely to present an acceptable behavior in a biological assay than a compound that has a high score from only a single prediction. As shown in Table 2, the predictions of computational hits were in agreement with the ones obtained in the in vivo assay as experimental hits. The five compounds that showed activity in the in vivo assay in general are shown in green, which means highly satisfactory results in the PHACA. Moreover, the compound that was inactive in vivo, due to its unsatisfactory drug-like properties, is shown in red. Taken together, compounds that show good PHACA results have a greater chance of being bioactive. We can also disregard molecules with poor predicted results. The findings showed that almost 50% of the compounds that were designed and synthesized were bioactive and showed good pharmacokinetic and pharmacodynamics properties alongside an acceptable toxicological profile.

### 2.6. Molecular Dynamics Studies of Compounds 6 and 9

**Figure 5:**
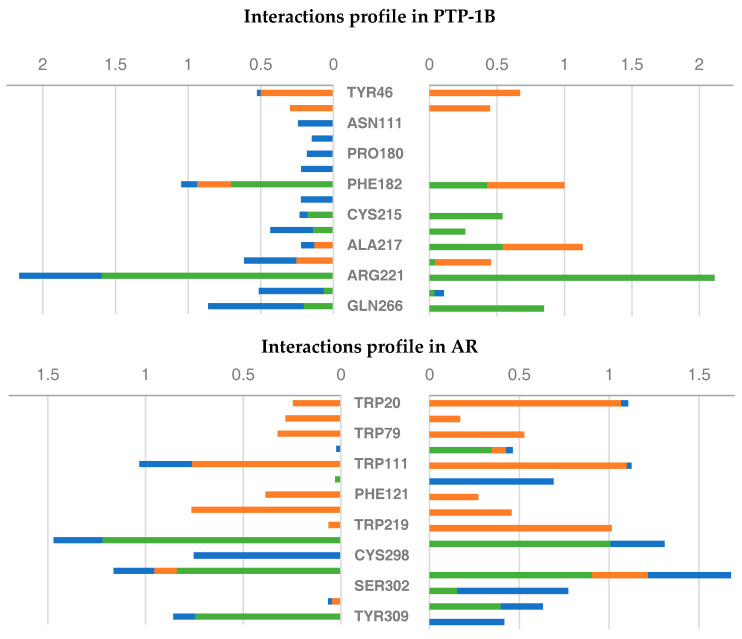
Protein–ligand interactions profile of Compounds **6** (left) and **9** (right) with PTP-1B and AR during the last 200 ns of simulation. The color of the bar represents the type of interaction (orange: hydrophobic; green: hydrogen-bond; blue: water-bridge). The fact that some residues can form more than one type of interaction allows the value to be larger than 1.

The previous results suggested two important points for bioactivity: (1) there are circa three atoms between the first aromatic ring and the acid functionality and (2) a phenyl electron-withdrawing substituent appears to decrease the activity. Thus, the most promising compounds (**6** and **9**) were analyzed through 300 ns of MD simulations, in order to analyze key features of the binding events. Relevant plots to the stability of simulation, such as protein and ligand RMSD are shown in Figure S2 (supplemental information), which suggested that the simulations were sufficiently converged to extract molecular insights. The flexibility of the targets was considered via the root-mean-square fluctuation profiles of the residues of AR and PTP-1B when bound to the crystallographic ligand and when bound to Compounds **6** and **9** (Figure S3). With respect to PTP-1B, the profile appears to be similar for the three compounds, with the exception of Residues 176–189, where the simulation with Compound **6** shows an increase in the fluctuations of those residues. Regarding AR, it is interesting to observe that there is a marked change in the region around Residues 218–230, which shows a decrease in the fluctuations on those residues when comparing simulations with Compounds **6** and **9** to the crystal structure. Taken together, these results suggest that the binding event with Compound **9** involves a decrease in the flexibility of the target. The interaction profile during the simulation for the two compounds was extracted and normalized over the last 200 ns to account for equilibration of the system (Figure 5). Regarding PTP-1B, the profile seems symmetrical for both compounds, interacting more frequently with Phe182 and Arg221, in a similar fashion to the crystallized inhibitor. However, there are some changes in the nature of the interactions: for Compound **6**, there are several contacts that possess an important component of water mediation, and these contacts are not present in Compound **9**. This suggests that Compound **6** is not optimally filling the whole binding cavity, thus allowing water molecules to mediate several key interactions for binding. Other subtle differences involve the change in the interactions with Phe182 for Compound **6** (more hydrogen-bond) and Compound **9** (hydrogen-bond/hydrophobic). Thus, this suggests that Compounds **6** and **9** have common, yet nonidentical binding determinants in PTP-1B. For AR, although Compounds **6** and **9** show similar and stable interactions when compared to the cocrystallized ligand, such as Trp111 and Leu300, other contacts (such as Thr113, Cys298, Ser302, and Cys303) suggest also that the binding modes of the two compounds are not the same.

**Figure 6:**
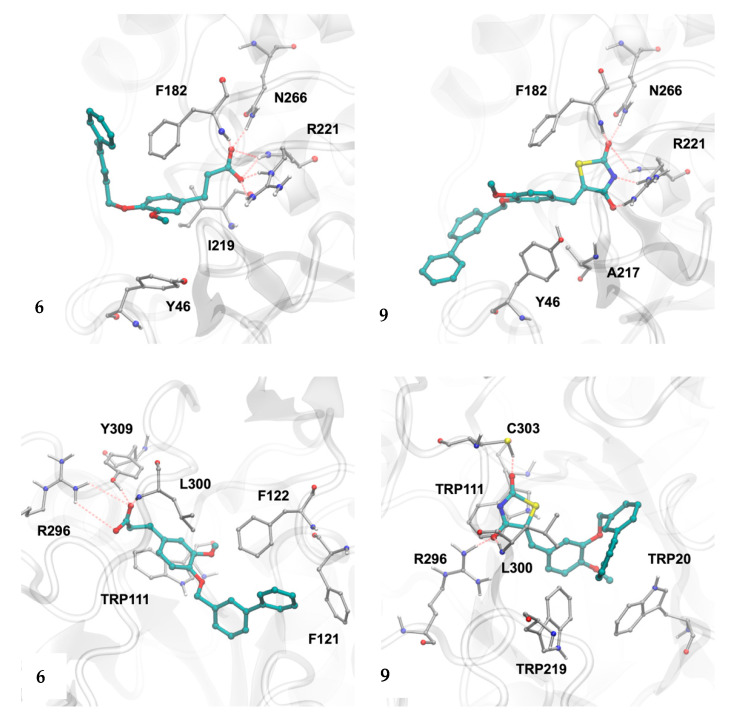
Most populated cluster for the simulations between Compounds **6** and **9** with PTP-1B (**top**) and AR (**bottom**) during the 300 ns of simulation. Hydrogen-bond interactions are indicated with dashed lines.

In order to obtain further insights in the binding mode of the compounds, visual analysis and a clustering of the trajectories were performed, and the most representative cluster for each simulation is shown in Figure 6. For PTP-1B, both Compounds **6** and **9** present a similar profile. Both compounds position the core aromatic ring for interaction with Phe182 and Tyr46, while the negative moiety interacts with Phe182, Arg221, and Asn266. There is no stabilization of the distal biphenyl moiety, except for the occasional interactions presented with Phe182, which forces this moiety to be mainly solvent-exposed. In the case of AR, Compound **6** locates the core aromatic ring for interaction with Trp111, with hydrogen-bond interactions with Arg296 and Tyr309, while the distal biphenyl moiety interacts evenly with Phe121 and Phe122. Frequent rotational movements were observed for the distal phenyl ring. In comparison, Compound **9** does not get as deep inside the binding pocket, positioning the thiazolidinedione moiety over Trp111. A rearrangement of Arg296 allows the hydrogen-bond interaction to be fulfilled for this compound, with further aid from Cys303. As the compound is not buried that deeply, the aromatic distal moieties interact with the farther tryptophan residues from the flexible loops and fix them closer to the binding pocket. Hence, Trp20 and Trp219 interact with the compounds, the nature of these contacts being mostly derived from π-stacking/hydrophobic interactions. In this binding mode, frequent rotational movements were also observed for the distal phenyl ring, equivalent due to its inherent symmetry.

The binding poses observed in MD also offer a plausible mechanism for the lack of activity of the other compounds. In the case of Compounds **1–3**, the shorter phenylacetic moiety would displace more internally the compound in AR, thus moving away the interactions with Phe121 and Phe122 and therefore decreasing the stability of the compound inside the binding pocket. In the case of compounds with the biphenyl-2-carbonitrile distal moiety, the breaking of symmetry regarding the rotation of the last aromatic ring would impair the interactions with Phe122 and Phe121 of the phenylacetic and phenylpropanoic heads, or the interactions with Trp219 and Trp20 of the thiazolidinedione heads. Although a more complete quantification of the binding events would require extensive sampling, the simulations suggest plausible binding modes and explain all of the experimental biological data. The simulations also offer support to the pharmacophore model, corroborating the ligand-based features with the structure-based features of PTP-1B and AR. In particular, Trp111 for AR and Phe182 for PTP-1B offer support for the first aromatic group, while Arg296/Cys303/Tyr309 for AR and Phe182/Arg221/Asn266 for PTP-1B act as the anchors for the negative charges, and Phe121/Phe122/Trp20/Trp219 finally stabilizes the distal moieties via hydrophobic interactions.

## 3. Materials and Methods

### 3.1. Chemistry

All of the reagents that we used are commercially available and were purchased from Sigma Aldrich^®^ (Saint Louis, MO, USA) or Merck^®^ (Darmstadt, Germany). ^1^H and ^13^C nuclear magnetic resonance spectra were obtained on a Variant Oxford Instrument (Palo Alto, CA, USA, 600 MHz and 150 MHz, respectively). DMSO-*d*_6_ was used as a deuterated solvent. Molecular masses were obtained with a JMS-700 spectrometer (JEOL, Tokyo, Japan) with an impact electronic method (70 eV). Melting points were obtained using an EZ-Melt MPA120 automated apparatus from Stanford Research Systems (Sunnyvale, CA, USA). Thin layer chromatography (TLC) was conducted on silica gel plates (Merck, Darmstadt, Germany).

#### 3.1.1. Procedure for the Synthesis of 3-(4-hydroxy-3-methoxyphenyl)propanoic acid (**11**)

Ferulic acid (1 g) was submitted to catalytic hydrogenation to reduce the double bond using a Shaker hydrogenation apparatus, Pd^0^/C (10%, 0.1 g), under a hydrogen atmosphere at 60 psi for 2 h, with EtOH as the solvent. The crude product was filtered in vacuo, and the filtrate was then immediately used without any further purification.

#### 3.1.2. General Procedure for the Synthesis of Compounds **1**–**6**

The compounds were synthesized using 2-(4-hydroxyphenyl)acetic acid (**10**) and 3-(4-hydroxy-3-methoxyphenyl)propanoic acid (**11**) as precursors. A mixture of 1.1 eq of the above compounds and 2.2 eq of K_2_CO_3_ was added to a round-bottom flask and were mechanically stirred using acetonitrile as the solvent. After 1 h, the respective arylmethylbromide or chloride (1 eq) was added to the mixture and was set in reflux to generate the respective substitution product under a nitrogen atmosphere. Once the reaction was completed, cold water and 10% aqueous HCl were added until a pH of 4.0 was reached, and a solid product was obtained. The crude product was filtered in vacuo. Recrystallization from the appropriate solvent was performed to afford colorless crystals of each compound.

[4-(1-naphthylmethoxy)phenyl]acetic acid (**1**)

The mixture of (4-hydroxyphenyl)acetic acid (**10**), K_2_CO_3_, and 1-(chloromethyl)naphthalene (**16**) was stirred and heated at 80 °C for 9 h. Recrystallized from EtOH, yield = 70%; mp = 114.8–116.3 °C; ^1^H-NMR (600 MHz, DMSO-d_6_) δ: 3.49 (s, 2H, CH_2_), 5.52 (s, 2H, OCH_2_), 7.04 (d, 2H, *Jo* = 8.64, H-3, H-5), 7.19 (d, 2H, *Jo* = 8.64, H-2, H-6), 7.51 (t, 1H, *Jo* = 8.1, H-3′), 7.54–7.59 (m, 2H, H-6, H-7), 7.67 (d, 1H, H-2′, *Jo* = 6.84), 7.93 (d, 1H, *Jo* = 8.22), 7.98 (d, 1H, *Jo* = 8.58), 8.09 (d, 1H, *Jo* = 8.04); ^13^C-NMR (150 MHz, DMSO-d_6_) δ: 40.49 (CH_2_), 68.23 (OCH_2_), 115.06 (C-3′, C-5′), 124.31 (C-8″), 125.8 (C-3″), 126.39 (C-6″), 126.8 (C-4″), 127.03 (C-7″). 127.8 (C-1′), 128.9 (C-2″), 129.06 (C-5″), 130.85 (C-2′, C-6′), 131.55 (C-4″a), 133.71 (C-8″a), 133.02 (C-1″), 157.62 (C-4′), 173.4 (C-1) ppm. EIMS: *m/z* = 292.3 [M^+^], 157.9 [M-135], 141.0 [M-151].

{4-[(2′-cyanobiphenyl-4-yl)methoxy]phenyl}acetic acid (**2**)

The mixture of (4-hydroxyphenyl)acetic acid (10), K_2_CO_3_, and 4′-bromomethyl-2-biphenylcarbonitrile (17) was stirred and heated at 80 °C for 15 h. Recrystallization from EtOH was performed to afford colorless crystals of **2**. Yield = 60%; mp = 164.9–166.8 °C; ^1^H-NMR (600 MHz, DMSO-d_6_) δ: 3.48 (s, 2H, CH_2_), 5.17 (s, 2H, OCH_2_), 6.99 (d, 2H, *Jo* = 8.58, H-3, H-5), 7.19 (d, 2H, *Jo* = 8.58, H-4, H-6), 7.59 (m, 5H, H-2′, H-6′, H-3′, H-5′, H-4″), 7.64 (d, 1H, Jo = 7.68, H-6″), 7.79 (t, 1H, *Jo* = 7.68, H-5″), 7.95 (d, 1H, *Jo* = 7.68, H-3″); ^13^C-NMR (150 MHz, DMSO-d_6_) δ: 39.68 (CH_2_), 68.74 (OCH_2_), 110.59 (C-2″’), 114.97 (C-3′, C-5′), 118.99 (C-4), 125.8 (C-3″), 126.39 (C-6″), 127.82 (C-6″’). 128.32 (C-3″, C-5″), 128.68 (C-4″’), 129.24 (C-2″, C-6″), 130.56 (C-1′), 130.86 (C-2′, C-6′), 133.98 (C-3″’), 134.24 (C-5″’), 137.68 (C-4″), 138.27 (C-1″), 144.65 (C-1″’), 157.48 (C-4′). 173.40 (C-1) ppm. EIMS: *m*/*z* = 343.3 [M^+^], 326 [M-17], 193.0 [M-151].

[4-(biphenyl-3-ylmethoxy)phenyl]acetic acid (**3**)

The mixture of (4-hydroxyphenyl)acetic (**10**) acid, K_2_CO_3_, and 3-phenylbenzyl bromide (**18**) was stirred and heated at 80 °C for 7 h. Recrystallization from EtOH was performed to afford crystals of **3**. Yield = 75%; mp = 141.9–143.8 °C. ^1^H-NMR (600 MHz, DMSO-d_6_) δ: 3.48 (s, 2H, CH_2_), 5.16 (s, 2H, OCH_2_), 6.98 (d, 2H, Jo = 8.58, H-3, H-5), 7.18 (d, 2H, Jo = 8.58, H-2, H-6), 7.37 (t, 1H, *Jo* = 7.56, H-5′), 7.44 (d, 1H, *Jo* = 7.56, H-4′), 7.49–7.46 (m, 3H, H-3″, H-4″, H-5″), 7.62 (d, 1H, *Jo* = 7.56, H-6′), 7.67 (d, 2H, *Jo* = 7.62, H-2″, H-6″), 7.72 (s, 1H, H-2′); ^13^C-NMR (150 MHz, DMSO-d_6_) δ: 39.68 (CH_2_), 68.74 (OCH_2_), 110.59 (C-2″’), 114.24 (C-3′, C-5′), 125.58 (C-2″), 125.76 (C-6″), 126.29 (C-4″’), 126.35 (C-2″’, C-6″’), 126.87 (C-5″), 127.17 (C-4″), 128.58 (C-3″’, C-5″’), 128.70 (C-1′). 130.02 (C-2′, C-6′), 137.54 (C-3″), 139.57 (C-1″), 139.95 (C-1″’), 172.55 (C-1). EIMS: *m*/*z* = 318.3 [M^+^], 241 [M-77], 165 [M-153].

3-[3-methoxy-4-(1-naphthylmethoxy)phenyl]propanoic acid (**4**)

The mixture of **11,** K_2_CO_3_, and 1-(chloromethyl)naphthalene (**16**) was stirred and heated for 8 h. Recrystallization from EtOH was performed to afford **4**. Yield = 64%; mp = 135.6–136.5 °C; ^1^H-NMR (600 MHz, DMSO-d_6_) δ: 2.52 (t, 2H, CH_2_), 2.77 (t, 2H, CH_2_), 3.72 (s, 3H, OCH_3_), 5.46 (s, 2H, OCH_2_), 6.74 (dd, 1H, H-6, *Jm* = 1.2, *Jo* = 8.4 Hz), 6.88 (s, 1H, H-2), 7.08 (d, 1H, H-5, *Jo* = 8.4 Hz), 7.50 (t, 1H, H-3′, *Jm* = 1.4, *Jo* = 7.8 Hz), 7.56 (m, 2H, H-6′, H-7′), 7.64 (d, 1H, H-2′, 7.2 Hz), 7.93 (d, 1H, H-5′, *Jo* = 8.4 Hz), 7.95 (d, 1H, H-4′, *Jo* = 9 Hz), 8.11 (d, 1H, H-8′, *Jo* = 7.8 Hz), 12.13 (s, COOH). ^13^C-NMR (150 MHz, DMSO-d6) δ: 30.03 (C-8, CH_2_), 35.5 (C-7, CH_2_), 55.4 (O-CH_3_), 68.6 (O-CH_2_), 112.57 (C-2), 114 (C-5), 119.9 (C-6), 124 (C-8′), 125.9 (C-3′), 126.3 (C-6′), 126.6 (C-7′), 128.2 (C-5′), 128.5 (C-4′), 131.1 (C-8a), 131.2 (C-1), 133.3 (C-1′, C-4a), 146.1(C-4), 149 (C-3), 173.8 (COOH). EIMS: *m*/*z* = 336.3 [M^+^], 156.0 [M-207], 127.0.

3-{4-[(2′-cyanobiphenyl-4-yl)methoxy]-3-methoxyphenyl}propanoic acid (**5**)

The mixture of **11** and 4′-bromomethyl-2-biphenylcarbonitrile (**17**) was stirred and heated at 80 °C for 2 h. Recrystallization from EtOH was performed to afford white crystals of **5**. Yield = 60%; mp = 133.7–134.9 °C; ^1^H-NMR (600 MHz, DMSO-d_6_) δ: 2.51 (t, 2H, CH_2_), 2.76 (t, 2H, H-7, CH_2_), 3.77 (s, 3H, OCH_3_), 5.12 (s, 2H, OCH_2_), 6.72 (dd, 1H, H-6, *Jm* = 1.8, *Jo* = 9.6 Hz), 6.80 (s, 1H, H-2), 6.96 (d, 1H, H-5, *Jo* = 7.8 HZ), 7.59 (m, 5H, H-2′, H-3′, H-5′, H-6′, H-4″), 7.64 (d, 1H, H-6″, *Jo* = 7.8 Hz), 7.95 (d, 1H, H-3″, *Jo* = 7.2 Hz). ^13^C-NMR (150 MHz, DMSO-d_6_) δ: 29.5 (C-7, CH_2_), 35.1 (C-8, CH_2_), 55.1 (C-10, OCH_3_), 69.2 (C-11 OCH_2_), 109.7 (C-2″), 112.2 (C-2), 117.3 (C-5, C-6″), 118.2 (C-12), 119.5 (C-6), 127.5 (C-2′, C-6″), 127.8 (C-4′), 128.3 (C-3′, C-5′), 129.7 (C-4″), 133.4 (C-1, C-3″), 133.6 (C-5″), 137.7 (C-1′), 143.8 (C-4), 145.7 (C-1″), 148.6 (C-3), 173.4 (C-9). EIMS: *m*/*z* = 387.4 [M^+^].

3-[4-(biphenyl-3-ylmethoxy)-3-methoxyphenyl]propanoic acid (**6**)

The mixture of **11** and 3-phenylbenzyl bromide (**18**) was stirred and heated at 80 °C for 12 h. Recrystallization from EtOH was performed to afford crystals of **6**. Yield = 84%; mp = 119.5–120.0 °C. ^1^H NMR (600 MHz, DMSO-d*6*) δ: 2.50 (t, 2H, CH_2_), 2.75 (t, 2H, CH_2_), 3.75 (s, 3H, OCH_3_), 5.11 (s, 2H, OCH_2_), 6.70 (dd, 1H, H-6, *Jm* = 1.8, *Jo* = 6.3 Hz), 6.87 (s, 1H, H-2), 6.95 (*d*, 1H, H-5, *Jo* = 7.8 Hz), 7.37 (t, 1H, H-5′, *Jo* = 7.2, *Jo* = 7.8 Hz), 7.42 (d, 1H, H-6′, *Jo* = 7.4 Hz), 7.47 (*t,* 3H, H-3″, H-4″, H-5″, *Jo* = 7.2, *Jo* = 7.8 Hz), 7.61 (*d*, 1H, H-4′, *Jo* = 7.8 Hz), 7.65 (*d*, 2H, H-2″, H-6″, *Jo* = 7.2 Hz) 12.09 (*s*, COOH). ^13^C-NMR (150 MHz, DMSO-d*6*) δ: 29.6 (C-8, CH_2_), 35.1 (C-7, CH_2_), 55.2 (OCH_3_), 69.7 (OCH_2_), 112.2(C-2), 113.5 (C-5), 119.6 (C-6), 126.3 (C-2′, C-4′), 127.2 (C-6′), 128.6 (C-3″, C-5″), 133.58 (C-1), 137.2 (C-2″, C-6″), 139.5 (C-1″, C-4″), 139.8 (C-1′,C-3′), 145.7 (C-4), 148.69 (C-3), 173.4 (COOH). EIMS: *m/z* = (% int. rel): 362.4 [M^+^].

#### 3.1.3. General Procedure for the Synthesis of Compounds **13**–**15**

The compounds were synthesized using isovanillin (**12**) as precursor. A mixture of 1.1 eq of **12** and 2.2 eq of K_2_CO_3_ was added to a round-bottom flask and was mechanically stirred using acetonitrile as a solvent. After 1 h, the respective arylmethylbromide or chloride (1 eq) was added to the mixture and was set in reflux under a nitrogen atmosphere to form the respective substitution product. The crude product was filtered in vacuo. Cold water was added to wash the crude product and was then filtered, thus obtaining a solid. Recrystallization from the appropriate solvent was performed to afford colorless crystals of each compound.

4-methoxy-3-(1-naphthylmethoxy)benzaldehyde (**13**)

The mixture of isovanillin and 1-(chloromethyl)naphthalene (**16**) was stirred and heated at 80 °C for 2 h. Recrystallization from EtOH was performed to afford colorless crystals of **13**. Yield = 81%; mp = 114.8–116.3 °C; ^1^H-NMR (600 MHz, DMSO-d_6_) δ: 3.92 (s, 3H, OCH_3_), 5.60 (s, 2H, OCH_2_), 7.01 (d, 1H, H-5, *Jo* = 8.3 Hz), 7.45 (t, 1H, H-3′, *Jo* = 7.2 Hz, *Jo* = 7.2 Hz), 7.49 (dd, 1H, H-7′, *Jm* = 1.8 Hz, *Jm* = 1.8 Hz, *Jo* = 8.2 Hz), 7.51–7.53 (m, 1H, H-6′, *Jm* = 1.2 Hz, *Jo* = 7.44 Hz), 7.55 (ddd, 1H, H-6, *Jm* = 1.5 Hz, *Jm* = 1.3 Hz, *Jm* = 1.3 Hz, *Jo* = 8.2 Hz, *Jo* = 6.8 Hz), 7.59 (d, 1H, H-2, *Jm* = 1.7 Hz), 7.62 (d, 1H, H-2′, *Jo* = 6.9 Hz), 7.84 (d, 1H, H-5′, *Jo* = 8.2 Hz), 7.88 (d, 1H, H-8′, *Jo* = 7.7 Hz), 8.08 (d, 1H, H-4′, *Jo* = 8.4 Hz), 9.84 (s, 1H, CHO). EIMS: *m*/*z* = 292.1 [M^+^].

4′−[(5-formyl-2-methoxyphenoxy)methyl]biphenyl-2-carbonitrile (**14**)

The mixture of isovainillin and 4′-bromomethyl-2-biphenylcarbonitrile (**17**) was stirred and heated at 80 °C for 4 h. Recrystallization from EtOH was performed to afford colorless crystals of **14**. Yield = 82%; mp = 157.4–158.3 °C; ^1^H-NMR (600 MHz, DMSO-d_6_) δ: 3.97 (s, 3H, OCH_3_), 5.24 (s, 2H, OCH_2_), 7.01 (d, 1H, H-5, *Jo* = 8.6 Hz), 7.43 (m, 1H, H-6, *Jo* = 7.7 Hz, *Jo* = 7.7 Hz), 7.49 (m, 3H, H-2, H-3″, H-4″, *Jm* = 1.6 Hz, *Jm* = 1.6 Hz, *Jm* = 1.6 Hz, *Jo* = 8.3 Hz), 7.58 (m, 4H, H-2′, H-3′, H-5′, H-6′, *Jo* = 8.8 Hz, *Jo* = 9 Hz), 7.63 (ddd, 1H, H-5″, *Jm* = 1 Hz, *Jm* = 1 Hz, *Jm* = 1 Hz, *Jo* = 7.7 Hz, *Jo* = 7.6 Hz), 7.76 (d, 1H, H-6″, *Jo* = 7.7 Hz), 9.84 (s, 1H, CHO). EIMS: *m*/*z* = 343.2 [M^+^].

3-(biphenyl-3-ylmethoxy)-4-methoxybenzaldehyde (**15**)

The mixture of isovanillin and 3-phenylbenzyl bromide (**18**) was stirred and heated at 80 °C for 2 h. Recrystallization from EtOH was performed to afford colorless crystals of **15**. Yield = 66%; mp = 82.1–83.9 °C. ^1^H-NMR (600 MHz, DMSO-d_6_) δ: 3.96 (s, 3H, OCH_3_), 5.25 (s, 2H, OCH_2_), 6.99 (d, 1H, H-5, *Jo* = 8.2 Hz), 7.35 (t, 1H, H-5′, *Jo* = 7.4 Hz, *Jo* = 7.4 Hz), 7.42–7.45 (m, 4H, H-2″, H-3″, H-5″, H-6″), 7.46–7.48 (m, 1H, H-6, *Jm* = 1.8 Hz, *Jm* = 1.9 Hz, *Jo* = 8.2 Hz), 7.49 (d, 1H, H-2, *Jm* = 1.7 Hz), 7.53–7.55 (m, 1H, H-4″), 7.59 (dd, 2H, H-4′, H-6′, *Jm* = 1 Hz, *Jm* = 1.2 Hz, *Jo* = 8.2 Hz), 7.68 (s, 1H, H-2′), 9.82 (s, 1H, CHO). EIMS: *m*/*z* = 318.1 [M^+^].

#### 3.1.4. General Procedure for the Synthesis of Compounds **7**–**9**

Compounds **13**–**15** (1 eq) were collocated in a round-bottom flask, and 1.1 eq of thiazolidine-2,4-dione was added. The mixture was stirred using toluene as the solvent and was then heated to reflux, after which 0.3 eq of piperidine and 0.3 eq of benzoic acid were added and coupled with a Dean–Stark system. The mixture was maintained in reflux for 4 h. The crude reaction was filtered in vacuo and was washed with water to afford the respective Compounds **7**–**9**.

(5 Z)-5-[4-methoxy-3-(1-naphthylmethoxy)benzylidene]-1,3-thiazolidine-2,4-dione (**7**)

General procedure was performed obtaining a yellow solid of **7**. Yield = 69%; m.p. = 233.0–234.9 °C; ^1^H-NMR (600 MHz, DMSO-d_6_) δ: 3.81 (s, 3H, OCH_3_), 5.59 (s, 2H, OCH_2_), 7.15–7.16 (d, 1H, H-5, *Jo* = 8.5 Hz), 7.21 (d, 1H, H-2′, *Jo* = 8.5 Hz), 7.43 (d, 1H, H-2, *Jm* = 1.7 Hz), 7.52 (t, 1H, H-3′, *Jo* = 8 Hz, *Jo* = 7.2 Hz), 7.56 (dd, 1H, H-7′, *Jm* = 1 Hz*, Jm* = 1.4 Hz, *Jo* = 7.1 Hz, *Jo* = 7.6 Hz), 7.59 (dd, 1H, H-6, *Jm* = 1.8 Hz, *Jm* = 1.1 Hz, *Jm* = 1.3 Hz, *Jo* = 8.2 Hz, *Jo* = 6.8 Hz), 7.67 (d, 1H, H-2′, *Jo* = 6.9 Hz), 7.75 (s, 1H, H-8,C=CH), 7.94 (d, 1H, H-5′, *Jo* = 8.22), 7.98 (dd, 1H, H-8′, *Jm* = 1.02, *Jo* = 7.95), 8.12 (d, 1H, H-4′, *Jo* = 8.2); ^13^C-NMR (150 MHz, DMSO-d_6_) δ: 55.74 (OCH_3_), 68.55 (OCH_2_), 112.44 (C-5), 115.53 (C-2), 120.59 (C-9), 123.88 (C-8′), 124.08 (C-3′), 125.36 (C-6), 125.7 (C-8), 125.98 (C-6′), 126.43 (C-1), 126.67 (C-4′), 128.44 (C-7′), 128.74 (C-2′), 131.11 (C-4′a), 132.03 (C-5′), 132.19 (C-1′), 133.26 (C-8′a), 147.89 (C-3), 151.24 (C-4), 167.36 (C-11), 167.93 (C-10). EIMS: *m*/*z*: 391.2 [M^+^].

4′-({5-[(Z)-(2,4-dioxo-1,3-thiazolidin-5-ylidene)methyl]-2-methoxyphenoxy}methyl) biphenyl-2-carbonitrile (**8**)

General procedure was performed obtaining a yellow solid of **8**. Yield = 63%; m.p. = 256.7–257.2 °C; ^1^H-NMR (600 MHz, DMSO-d6) δ: 3.83 (s, 3H, OCH_3_), 5.21 (s, 2H, OCH_2_), 7.12–7.14 (d, 1H, H-5, *Jo* = 8.4 Hz), 7.17–7.19 (m, 1H, H-6, *Jm* = 1.8 Hz, *Jm* = 1.8 Hz, *Jo* = 8.5 Hz), 7.26–7.27 (d, 1H, H-2, *Jm* = 1.8 Hz), 7.55–7.57 (ddd, 1H, H-4″, *Jm* = 1 Hz, *Jm* = 1 Hz, *Jm* = 1 Hz, *Jo* = 7.7 Hz, *Jo* = 7.6 Hz), 7.58–7.61 (m, 4H, H-2′, H-3′, H-5′, H-6′, *Jo* = 8.4 Hz, *Jo* = 7.6 Hz), 7.71–7.72 (d, 1H, H-6″, *Jo* = 7.3 Hz), 7.65 (s, 1H, H-8, C=CH), 7.75–7.78 (ddd, 1H, H-5″, *Jm* = 1.2 Hz, *Jm* = 1.1 Hz, *Jm* = 1.1 Hz, *Jo* = 7.7 Hz, *Jo* = 7.7 Hz), 7.92–7.93 (dd, 1H, H-3″, *Jm* = 1 Hz, *Jo* = 7.4 Hz). ^13^C-NMR (150 MHz, DMSO-d6) δ: 56.22 (OCH_3_), 69.98 (OCH_2_), 110.61 (C-2″), 112.85 (C-5), 115.31 (C-2), 124.49 (C-6), 126.38 (C-8), 128.54 (C-2′, C-6′), 128.69 (C-6″), 129.27 (C-5′, C-3′), 130.56 (C-4″), 131.43 (C-1), 133.96 (C-3″), 134.27 (C-5″), 137.78 (C-1″), 137.88 (C-4′), 144.63 (C-1′), 148.25 (C-3), 151.38 (C-4), 169.22 (C-11), 169.45 (C-10). EIMS, *m*/*z*: 442.2 [M^+^].

(5 Z)-5-[3-(biphenyl-3-ylmethoxy)-4-methoxybenzylidene]-1,3-thiazolidine-2,4-dione (**9**)

General procedure was performed obtaining a yellow solid of **9**. Yield = 55%; m.p. = 202.1–206.2 °C; ^1^H-NMR (600 MHz, DMSO-d6) δ: 3.85 (s, 3H, OCH_3_), 5.23 (s, 2H, OCH_2_), 7.14–7.15 (d, 1H, H-5, *Jo* = 8.5 Hz), 7.20–7.21 (m, 1H, H-6, *Jm* = 1.5 Hz, *Jm* = 1.6 Hz, *Jo* = 8.5 Hz), 7.27–7.28 (d, 1H, H-2, *Jm* = 1.9 Hz), 7.36–7.39 (m, 1H, H-5′, *Jo* = 7.4 Hz, *Jo* = 7.4 Hz), 7.45–7.50 (m, 4H, H-2″, H-3″, H-5″, H-6″), 7.62–7.63 (d, 1H, H-4″, *Jo* = 7.6 Hz), 7.66–7.67 (m, 2H, H-4′, H-6′, *Jm* = 1.1 Hz, *Jo* = 7.8 Hz), 7.71 (s, 1H, H-8), 7.76 (s, 1H, H-2′); ^13^C-NMR (150 MHz, DMSO-d6) δ: 55.77 (OCH_3_), 69.96 (OCH_2_), 112.41 (C-5), 114.87 (C-2), 120.69 (C-9), 124.41 (C-4′), 125.57 (C-6), 126.14 (C-8), 126.29 (C-2′), 126.69 (C-2″, C-6″), 126.79 (C-1), 127.54 (C-5′), 128.93 (C-3″, C-5″), 129.11 (C-4″), 131.97 (C-6′), 137.41 (C-1′), 139.88 (C-1″), 140.35 (C-3′), 147.81 (C-3), 151.19 (C-4), 167.40 (C-11), 167.92 (C-10). EIMS, *m*/*z*: 417.4 [M^+^].

### 3.2. Animals

ICR male mice weighing 25 ± 5 g were housed in animal cages with 12 h periods of light and 12 h periods of dark. Animals were allowed to acclimatize to the laboratory conditions. The animals were maintained in a 25 °C environment, and water and food were provided ad libitum. All animal experiments were conducted according to protocols that were approved by the Mexican government NOM-065-ZOO-1999 and NOM-033-ZOO-2014 [33,34].

#### 3.2.1. Induction of Diabetes

Induction of diabetes was performed according to previous works [35,36]. Animals were fasted for 8 h. Nicotinamide (NA) was dissolved in sterile water and administered at a dose of 20 mg/kg i.p. Fifteen minutes later, streptozotocin (STZ) was administered at a dose of 100 mg/kg i.p. (citrate buffer 0.05 M, pH = 4.5). The mice that presented with blood glucose levels over 150 mg/dL were used for the in vivo assay.

#### 3.2.2. Acute Antidiabetic Assay

We performed this assay according to a previous methodology that was used in our lab [5,37]. Briefly, the animals were fasted for 8 h, after which the groups (n = 6) were administered a 100 mg/kg dose of the different treatments, in addition to one vehicle group (10% Tween 80) and one positive control group (20 mg/kg glibenclamide). Blood glucose levels were measured at 0, 1, 3, 5, and 7 h by a commercial glucometer (Accu-Check Performa).

### 3.3. Crystal Structures

The crystallographic data of the proteins PTP-1B and AR were downloaded from PDB (4Y14 [38] and 4XZH [39], respectively). Both of these enzymes were cocrystallized along their specific inhibitors (i.e., bound to compounds CPT157633 and JF0048, respectively). The structures were manually curated using Maestro 12.1 with the Protein Preparation Wizard from the Schrodinger suite 2019-4 (Schrödinger LLC, 2019, New York, NY, USA). After removal of unnecessary molecular entities in each structure such as ions, cosolvents, and water molecules, the hydrogen-bond network and rotameric conformations were optimized, and a restrained minimization was performed.

### 3.4. Molecular Dynamics Simulations

MD studies of the protein–ligand complexes were performed using Desmond (version 2019-4, Schrödinger, New York, NY, USA) with the OPLS3e forcefield [40]. The complexes were prepared with the System Builder Utility in a 10 Å buffered rhombic dodecahedron box, using the transferable intermolecular potential with a 3-point model for water (TIP3P). The complexes were neutralized, and NaCl was added in a 0.15 M concentration. Complexes were minimized using the steep-descent (SD) algorithm followed by the Broyden–Fletcher–Gold–Farb–Shanno (LBFGS) method in three stages. In the first stage, water heavy atoms were restrained with a force constant of 1000 kcal mol^−1^ Å^−2^ for 5000 steps (1000 SD, 4000 LBFGS) with a convergence criterion of 50 kcal mol^−1^ Å^−2^; for the second stage, backbones were constrained with a 10 kcal mol^−1^ Å^−2^ force constant using a convergence criterion of 10 kcal mol^−1^ Å^−2^ for 2000 steps (1000 SD, 1000 LBFGS); for the third stage, the systems were minimized with no restraints for 1000 steps (750 SD, 250 LBFGS) with a convergence criterion of 1 kcal mol^−1^ Å^−2^. Equilibration was carried out in several steps. At first, Brownian dynamics for 250 ps were determined with the Berendsen thermostat. Simulation on the NVT ensemble was then performed, slowly heating from 10 to 300 K over 3000 ps. At this stage, constraints were enforced on solute heavy atoms, using a constant of 50 kcal mol^−1^. Finally, equilibration on NPT ensemble used the Berendsen thermostat and Langevin barostat for additional 250 ps was done.

With the system ready for production, 300 ns of simulation were performed, under an NPT ensemble at 1 bar and 300 K using the Martyna–Tuckerman–Klein (MTK) barostat and the Nosé–Hoover thermostat. Electrostatic forces were calculated with the smooth PME method using a 9 Å cut-off, while constraints were enforced with the M-SHAKE algorithm. Integration was done every 2 fs, with a recording interval of 500 ps. The quality analysis of the simulation and other trajectory analyses were carried out with the tools implemented in the Maestro 12.1 (Schrödinger, New York, NY, USA) and with VMD [41].

### 3.5. Ensemble Docking

The simulations of the two receptors in the *holo* state (i.e., drug-bound) were clustered based on the backbone and sidechain RMSD, and the first six clusters, covering ~60% of the sampled conformational space, were recovered. The obtained conformations were stripped from waters and ions and subjected to a restrained minimization of the side chains. With the nine compounds, and the corresponding cocrystallized inhibitor, docking procedures were performed on the six protein conformations. All docking procedures were performed with Glide 8.4 with the XP methodology [42] and the OPLS3e forcefield [40]. A grid was created around the binding pocket in a box of 25 Å of length per side, in order to allow the ligand to explore diverse binding poses around the pocket.

After the docking calculations were performed for the six protein conformations, the results for each individual compound were pooled and clustered following the ligand heavy atoms. For data fusion in ensemble docking, multiple approaches have been proposed [43]. For our study, a simple data fusion approach was followed: from the clustering of the docked poses, the pose within the most populated cluster was selected. For assigning the docking score, the lowest value within the most populated cluster was chosen, in order to reflect the best achieved score.

From the total number of poses, the percentage of poses that were included in the top cluster was also included in the report table. Validation of the docking protocol used was carried out by redocking the respective cocrystallized ligands. It was observed that the cocrystallized inhibitor docked adequately in the six conformers (not more than 2.0 Å of RMSD) and that the clustering of all poses also generated a most-populated cluster with the correct crystallographic pose within the 2.0 Å of tolerance.

### 3.6. Pharmacological Consensus Analysis

An a priori pharmacological consensus analysis (PHACA) was performed with the results that were obtained from several computational tools. The pharmacodynamics properties were calculated from the output score that was obtained with Glide XP. The following pharmacokinetic properties were acquired using AdmetSAR and SwissADME software: consensus logP, water solubility class, human intestinal absorption, blood–brain permeability, P-glycoprotein substrate, bioavailability score, and inhibition of CYP3A4. Toxicological profiles (Ames, hERG channel blockage, and carcinogenesis) were obtained from the web server AdmetSAR and ACD/Tox Suite version 2.95 [22,23,44]. To visualize the pharmacological consensus analysis, we used a color code indicating chances that the compound has drug-like properties, as follows: green (very satisfactory), yellow (satisfactory), and red (unsatisfactory). We considered three properties for each of the points. Depending on the number of properties they satisfied, we classified them with the respective color mentioned above. These colors were assigned in reference to other work [45].

### 3.7. Statistical Analysis

The data were analyzed by two-way ANOVA followed by Dunnett’s post hoc test. The results are expressed as the means ± S.E.M. Statically significance was assumed when *p* values were <0.05.

## 4. Conclusions

In this work, we reported the design, synthesis, and in vivo evaluation of nine compounds that were developed for the experimental treatment of diabetes, and we aimed to complement a unified antidiabetic pharmacophore with multitarget action. An a priori analysis of the compounds employing a pharmacological consensus analysis (PHACA) showed highly satisfactory results for five compounds, and their in vivo evaluation confirmed their biological activity. The molecules controlled the blood glucose levels without exacerbating the effect, in contrast to glibenclamide, which had a robust hypoglycemic effect. Moreover, molecular dynamics analysis shed light on the molecular recognition process and provided an explanation for the experimental results obtained. Together, these findings provide a rationale for the molecular design we employed in our lab, as we found the unified pharmacophore to possess a strong antidiabetic activity. Moreover, the use of an acid bioisostere surrogate, such as thiazolidine-2,4-dione or carboxylic acid, resulted in an interaction similar to those presented by the original cocrystallized inhibitors. Indeed, Compounds **6** and **9** are interesting bioactive compounds for the treatment of diabetes due to their antihyperglycemic effect. Further studies are needed to identify other possible targets in the body [46], in order to fully characterize its polypharmacological profile.

## Figures and Tables

**Table 1:** Percentage of variation of blood glucose concentration during the in vivo acute antidiabetic assay performed with Compounds **1–9**.

Compound	Time Course and % of Variation of Glycemia			
	1 h	3 h	5 h	7 h
1	44.4	35.9	30.5	8.2
2	−0.2 *	−22.8 *	−24.5	−21.6
3	16.3	−8.4	−13.7	−17.3
4	14.7	7.9	−2.7	−10.4
5	24.7	−21.1 *	−14.1	−16.1
6	7.1 *	−18.5 *	−18.5	−30.0
7	20.6	19.7	−1.6	−8.8
8	−3.1 *	−5.9	−8.1	−31.7
9	3.2 *	−10.7 *	−23.4	−30.9
Control	52.9	15.3	0.2	−11.5
Glibenclamide	−12.8 *	−33.9 *	−42.6 *	−64.6 *

* Statistically significant versus control group, two-way ANOVA, comparison by Dunnett’s test (n = 6, mean ± SEM, *p* < 0.05).

**Table 2:** Color code pharmacological consensus analysis (PHACA) for the evaluated compounds.

Property/Compound	1	2	3	4	5	6	7	8	9
**Physicochemical properties**									
**Log P**									
**Predicted solubility**									
**Lipinski Ro5**									
**Pharmacodynamics properties (Molecular docking)**									
**PTP-1B**									
**AR**									
**Pharmacokinetic properties**									
**Absorption**									
**PGp substrate**									
**CYP1A2 inhibitor**									
**CYP2D6 inhibitor**									
**CYP3A4 Inhibitor**									
**Toxicity prediction**									
**Mutagenicity**									
**Carcinogenicity**									
**hERG blockage**									
**Experimental antihyperglycemic data for analogue compounds** [5,17]									
**In vivo assay**	**No**	**No**	**No**	**No**	**Active**	**Active**	**No**	**Active**	**Active**
**Overall Score**									
**Predicted analysis result**									
**Current experimental result**									

: green = very satisfactory;             : yellow = satisfactory;             : red = unsatisfactory.

## Data Availability

No other data were reported.

## Supplementary Materials

Supplementary materials can be found. Figure S1: Interactions profile for the co-crystallized compounds during the 300 ns simulation for PTP-1B (left) and AR (right), Figure S2: Protein and ligand RMSD profiles for compounds **6** and **9** during the 300 ns simulations. The large fluctuations on the ligand RMSD are due to the flipping of the distal biphenyl moiety, which in PTP-1B is solvent-exposed, and in AR is stabilized by two contiguous residues, Figure S3: Root mean square fluctuations of the carbon alpha for the two targets during the simulations with the crystal inhibitor and compounds **6** and **9**, Table S1: Ligand interactions in PTP-1B and AR. Score values under −6.0 kcal/mol were considered good values. Residues also involved in the interaction with the co-crystallized inhibitor are marked in red. The percentage of conformations in the largest cluster is reported in cluster size, while the most negative value is reported for the score, Table S2: Calculated pharmacokinetic and genotoxic properties for compounds **1**–**9**, Table S3: Acute toxicity profile predicted for compounds **1**–**9**, pioglitazone and glibenclamide.

## Sample Availability

Samples of the compounds are available from the authors.

## References

[1] International Diabetes Federation (2019). IDF Diabetes Atlas 2019.

[2] Lemanowicz A., Leszczyński W., Rusak G., Bialecki M., Ratajczak P. (2018). Chest Adipose Tissue Distribution in Patients with Morbid Obesity. Pol. J. Radiol..

[3] Safavi M., Forolumadi A., Abdollahi M. (2013). The Importance of Synthetic Drugs for Type 2 Diabetes Drug Discovery. Expert Opin. Drug Discov..

[4] Inzucchi S.E., Bergenstal R.M., Buse J.B., Diamant M., Ferranini E., Nauck M., Peters A.L., Tsapas A., Wender R., Matthews D.R. (2015). Management of Hyperglycemia in Type 2 Diabetes: A Patient-Centered Approach. Diabetes Care.

[5] Colín-Lozano B., Estrada-Soto S., Chávez-Silva F., Gutiérrez-Hernández A., Cerón-Romero L., Giacoman-Martínez A., Almanza-Pérez J.C., Hernández-Núñez E., Wang Z., Xie X. (2018). Design, Synthesis and in Combo Antidiabetic Bioevaluation of Multitarget Phenylpropanoic Acids. Molecules.

[6] Saidu Y., Muhammad S.A., Abbas A.Y., Onu A., Tsado I.M., Muhammad L. (2017). In vitro screening for protein tyrosine phosphatase 1B and dipeptidyl peptidase IV inhibitors from selected Nigerian medicinal plants. J. Intercult. Ethnopharmacol..

[7] Maccari R., Ottanà R. (2015). Targeting aldose reductase for the treatment of diabetes complications and inflammatory diseases: New insights and future directions. J. Med. Chem..

[8] He J., Gao H.-X., Yang N., Zhu X.-D., Sun R.-B., Xie Y., Zheng C.-H., Zhang J.-W., Wang J.-K., Ding F. (2019). The aldose reductase inhibitor epalrestat exerts nephritic protection on diabetic nephropathy in db/db mice through metabolic modulation. Acta Pharmacol. Sin..

[9] Herrera-Rueda M.A., Tlahuext-Romero H., Paoli P., Giacoman-Martínez A., Almanza-Pérez J.C., Pérez-Sánchez H., Gutiérrez-Hernández A., Chávez-Silva F., Domínguez-Mendoza E.A., Estrada-Soto S. (2018). Design, synthesis, in vitro, in vivo and in silico pharmacological characterization of antidiabetic *N*-Boc-L-tyrosine-based compounds. Biomed. Pharmacother..

[10] Hidalgo-Figueroa S., Navarrete-Vázquez G., Estrada-Soto S., Giles-Rivas D., Alarcón-Aguilar F.J., León-Rivera I., Giacoman-Martínez A., Miranda Pérez E., Almanza-Pérez J.C. (2017). Discovery of New Dual PPARγ-GPR40 Agonists with Robust Antidiabetic Activity: Design, Synthesis and in Combo Drug Evaluation. Biomed. Pharmacother..

[11] Maccari R., Del Corso A., Paoli P., Adornato I., Lori G., Balestri F., Cappiello M., Naß A., Wolber G., Ottanà R. (2018). An investigation on 4-thiazolidinone derivatives as dual inhibitors of aldose reductase and protein tyrosine phosphatase 1B, in the search for potential agents for the treatment of type 2 diabetes mellitus and its complications. Bioorg Med. Chem. Lett..

[12] Bialy L., Waldman H. (2005). Inhibitors of Protein Tyrosine Phosphatases: Next-Generation Drug. Angew. Chem. Int. Edit..

[13] Hidalgo-Figueroa S., Estrada-Soto S., Paoli P., Lori G., León-Rivera I., Navarrete-Vázquez G. (2018). Synthesis and evaluation of thiazolidine-2,4-dione/benzazole derivatives as inhibitors of protein tyrosine phosphatase 1B (PTP-1B): Antihyperglycemic activity with molecular docking study. Biomed. Pharmacother..

[14] Karplus M., Kuriyan J. (2005). Molecular dynamics and protein function. Proc. Natl. Acad. Sci. USA.

[15] Roy U. (2019). 3D Modeling of Tumor Necrosis Factor Receptor and Tumor Necrosis Factor-bound Receptor Systems. Mol. Inform..

[16] Roy U. (2017). Structural modeling of tumor necrosis factor: A protein of immunological importance. Biotechnol. Appl. Biochem..

[17] Fu Y., Liu Y.-X., Yi K.-H., Li M.-Q., Li J.-Z., Ye F. (2019). Quantitative Structure Activity Relationship Studies and Molecular Dynamics Simulations of 2-(Aryloxyacetyl)cyclohexane-1,3-Diones Derivatives as 4-Hydroxyphenylpyruvate Dioxygenase Inhibitors. Front. Chem..

[18] Decherchi S., Cavalli A. (2020). Thermodynamics and Kinetics of Drug-Target Binding by Molecular Simulation. Chem. Rev..

[19] Rastelli G., Costantino L. (1998). Molecular dynamics simulations of the structure of aldose reductase complexed with the inhibitor tolrestat. Bioorg Med. Chem. Lett..

[20] Johnson T.O., Ermolieff J., Jirousek M.R. (2002). Protein tyrosine phosphatase 1B inhibitors for diabetes. Nat. Rev. Drug Discov..

[21] Cheng F., Li W., Zhou Y., Shen J., Wu Z., Liu G., Lee P.W., Tang Y. (2012). Correction to “admetSAR: A Comprehensive Source and Free Tool for Assessment of Chemical ADMET Properties”. J. Chem. Inf. Model..

[22] Daina A., Michielin O., Zoete V. (2017). SwissADME: A free web tool to evaluate pharmacokinetics, drug-likeness and medicinal chemistry friendliness of small molecules. Sci. Rep..

[23] Hidalgo-Figueroa S., Ramírez-Espinosa J.J., Estrada-Soto S., Almanza-Pérez J.C., Román-Ramos R., Alarcón-Aguilar F.J., Hernández-Rosado J.V., Moreno-Díaz H., Díaz-Coutiño D., Navarrete-Vazquez G. (2013). Discovery of thiazolidine-2,4-dione/biphenylcarbonitrile Hybrid as Dual PPAR α/γ Modulator With Antidiabetic Effect: In Vitro, in Silico and in Vivo Approaches. Chem. Biol. Drug. Des..

[24] Pharaon L.F., El-Orabi N.F., Kunhi M., Al-Yacoub N., Awad S.M., Poizat C. (2017). Rosiglitazone promotes cardiac hypertrophy and alters chromatin remodeling in isolated cardiomyocytes. Toxicol. Lett..

[25] Yki-Jarvinen H. (2004). Thiazolidinediones. N. Engl. J. Med..

[26] Yaghoubi M., Jafari S., Sajedi B., Gohari S., Akbarieh S., Heydari A.H., Jameshoorani M. (2017). Comparison of fenofibrate and pioglitazone effects on patients with nonalcoholic fatty liver disease. Eur. J. Gastroenterol. Hepatol..

[27] Skochko O.V., Kaidashev I.P. (2017). Effect of Pioglitazone on Insulin Resistance, Progression of Atherosclerosis and Clinical Course of Coronary Heart Disease. Wiad. Lek..

[28] American Diabetes Association, Diabetes Type 2. http://www.diabetes.org/diabetes-basics/type-2.

[29] Numonov S., Edirs S., Bobakulov K., Nasimullah M.Q., Bozorov K., Sharopov F., Setzer W.N., Zhao H., Habasi M., Sharofova M. (2017). Evaluation of the antidiabetic activity and chemical composition of Geranium collinum root extracts—Computational and experimental investigations. Molecules.

[30] Rotinsulu H., Yamazaki H., Sugai S., Iwakura N., Wewengkang D.S., Sumilat D.A., Namikoshi M. (2018). Cladosporamide A, a New Protein Tyrosine Phosphatase 1B Inhibitor, Produced by an Indonesian Marine Sponge-Derived *Cladosporium* Sp.. J. Nat. Med..

[31] Klaman L.D., Boss O., Peroni O.D., Kim J.K., Martino J.L., Zabolotny J.M., Moghal N., Lubkin M., Kim Y.-B., Sharpe A.H. (2000). Regulation of Insulin-Like Growth Factor Type I (IGF-I) Receptor Kinase Activity by Protein Tyrosine Phosphatase 1B (PTP-1B) and Enhanced IGF-I-Mediated Suppression of Apoptosis and Motility in PTP-1B-Deficient Fibroblasts. Mol. Cell. Biol..

[32] Chhajed S.S., Chaskar S., Kshirsagar S.K., Haldar G.M., Kar Mahapatra D. (2017). Rational Design and Synthesis of Some PPAR-γ Agonists: Substituted benzylideneamino-benzylidene-thiazolidine-2,4-diones. Comput. Biol. Chem..

[33] Servicio Nacional de Sanidad, Inocuidad y Calidad Agroalimentaria, Gobierno de México, Norma Oficial Mexicana: NOM-062-ZOO-1999 Especificaciones técnicas para la producción, cuidado y uso de los animales de laboratorio. https://www.gob.mx/senasica/documentos/nom-062-zoo-1999.

[34] Procuraduría Federal de Protección al Ambiente, Gobierno de México, Norma oficial Mexicana: NOM-033-SAG/ZOO-20, Métodos para dar muerte a los animales domésticos y silvestres. https://www.gob.mx/profepa/documentos/norma-oficial-mexicana-nom-033-sag-zoo-2014-metodos-para-dar-muerte-a-los-animales-domesticos-y-silvestres.

[35] Guzmán-Ávila R., Flores-Morales V., Paoli P., Camici G., Ramírez-Espinosa J.J., Cerón-Romero L., Navarrete-Vázquez G., Hidalgo-Figueroa S., Ríos M.Y., Villalobos-Molina R. (2018). Ursolic acid derivatives as potential antidiabetic agents: In vitro, in vivo, and in silico studies. Drug Dev. Res..

[36] Tahara A., Matsuyama-Yokono A., Shibasaki M. (2011). Effects of Antidiabetic Drugs in High-Fat Diet and Streptozotocin-Nicotinamide-Induced Type 2 Diabetic Mice. Eur. J. Pharmacol..

[37] Gutierrez-Hernández A., Galván-Ciprés Y., Domínguez-Mendoza E.A., Aguirre-Vidal Y., Estrada-Soto S., Almanza-Pérez J.C., Navarrete-Vázquez G. (2019). Design, Synthesis, Antihyperglycemic Studies, and Docking Simulations of Benzimidazole-Thiazolidinedione Hybrids. J. Chem..

[38] Krishnan N., Krishnan K., Connors C.R., Choy M.S., Page R., Peti W., Van Aelst L., Shea S.D., Tonks N.K. (2015). PTP1B inhibition suggests a therapeutic strategy for Rett syndrome. J. Clin. Investig..

[39] Ruiz F.X., Cousido-Siah A., Porté S., Domínguez M., Crespo I., Rechlin C., Mitschler A., de Lera Á.R., Martín M.J., de la Fuente J.Á. (2015). Structural Determinants of the Selectivity of 3-Benzyluracil-1-acetic Acids toward Human Enzymes Aldose Reductase and AKR1B10. ChemMedChem.

[40] Roos K., Wu C., Damm W., Reboul M., Stevenson J.M., Lu C., Dahlgren M.K., Mondal S., Chen W., Wang L. (2019). OPLS3e: Extending Force Field Coverage for Drug-Like Small Molecules. J. Chem. Theory Comput..

[41] Humphrey W., Dalke A., Schulten K. (1996). VMD—Visual Molecular Dynamics. J. Mol. Graphics.

[42] Friesner R.A., Murphy R.B., Repasky M.P., Frye L.L., Greenwood J.R., Halgren T.A., Sanschagrin P.C., Mainz D.T. (2006). Extra Precision Glide: Docking and Scoring Incorporating a Model of Hydrophobic Enclosure for Protein-Ligand Complexes. J. Med. Chem..

[43] Bajusz D., Rácz A., Héberger K. (2019). Comparison of Data Fusion Methods as Consensus Scores for Ensemble Docking. Molecules.

[44] Gutierrez-Lara E., Martínez-Conde C., Rosales-Ortega E., Ramírez-Espinosa J.J., Rivera-Leyva J.C., Centurión D., Carvajal K., Ortega-Cuellar D., Estrada-Soto S., Navarrete-Vázquez G. (2017). Synthesis and In Vitro AMPK Activation of Cycloalkyl/Alkarylbiguanides with Robust In Vivo Antihyperglycemic Action. J. Chem..

[45] Khan T., Ahmad R., Azad I., Raza S., Joshi S., Khan A.R. (2018). Computer-aided drug design and virtual screening of targeted combinatorial libraries of mixed-ligand transition metal complexes of 2-butanone thiosemicarbazone. Comput. Biol. Chem..

[46] Ramírez-Nava E.J., Hernández-Ochoa B., Navarrete-Vázquez G., Arreguín-Espinosa R., Ortega-Cuellar D., González-Valdez A., Martínez-Rosas V., Morales-Luna L., Martínez-Miranda J., Sierra-Palacios E. (2021). Novel inhibitors of human glucose-6-phosphate dehydrogenase (HsG6PD) affect the activity and stability of the protein. Biochim. Biophys. Acta Gen. Subj..

