# Supplementary material for: Design, Synthesis, and In Silico Multitarget Pharmacological Simulations of Acid Bioisosteres with a Validated In Vivo Antihyperglycemic Effect
Source: Molecules. 2021 Feb 4;26(4):799. doi: 10.3390/molecules26040799 (PMC7913794; doi:10.3390/molecules26040799)
Supplement: Supplementary file 1 [file molecules-26-00799-s001.pdf]

Supporting information

Design, synthesis and *in silico* multi-target pharmacological simulations of acid bioisosteres with validated *in vivo* antihyperglycemic effect

Elix Alberto Domínguez-Mendoza,[1] Yelzyn Galván-Ciprés,[1] Josué Martínez-Miranda,[1] Cristian Miranda-González,[1] Blanca Colín-Lozano,[1] Emanuel Hernández-Núñez,[2] Gloria Hernández-Bolio,[2] Oscar Palomino-Hernández,[3,4] and Gabriel Navarrete-Vazquez[1,*]

[1] Facultad de Farmacia, Universidad Autónoma del Estado de Morelos, Cuernavaca, Morelos 62209, México; ElixRose@outlook.com (E.A. D.-M.); yelzyn.galvanc@uaem.edu.mx (Y.G.-C.); josue.martinezm@uaem.edu.mx (J.M.-M.); cristian.mirandag@uaem.edu.mx (C..M.-G.); clbi_ff@uaem.mx (B.C.-L.);

[2] Cátedra CONACyT, Departamento de Recursos del Mar, Centro de Investigación y de Estudios Avanzados del IPN, Unidad Mérida, 97310, Yucatán, México; emanuel.hernandez@cinvestav.mx (E.H.-N.)

[3] Computational Biomedicine (IAS-5/INM-9), Forschungszentrum Juelich, 52425, Jülich, Germany; o.palomino@fz-juelich.de

[4] Department of Chemistry, Rheinisch-Westfälische Technische Hochschule Aachen, 52425 Aachen, Germany;

* Correspondence e-mail: gabriel_navarrete@uaem.mx; Tel.: +52-777-329-7089

---

§Taken in part from the postdoctoral stay of E. A. Dominguez-Mendoza.

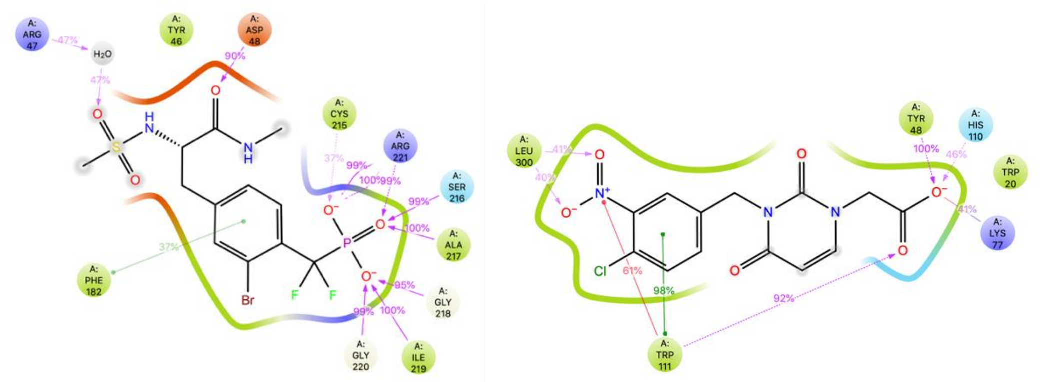

Figure S1. Interactions profile for the co-crystallized compounds during the 300 ns simulation for PTP-1B (left) and AR (right).

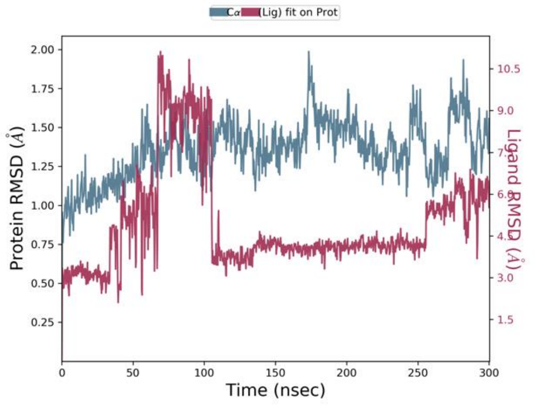

Prop, 6, PTP-1B

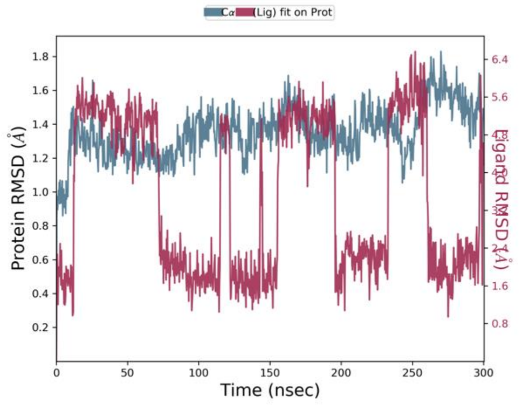

Thia, 9, PTP-1B

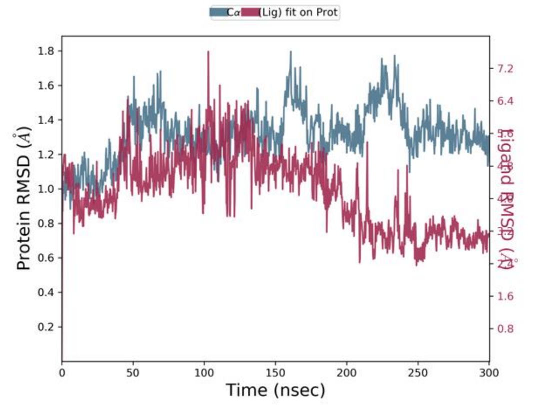

Prop, 6, AR

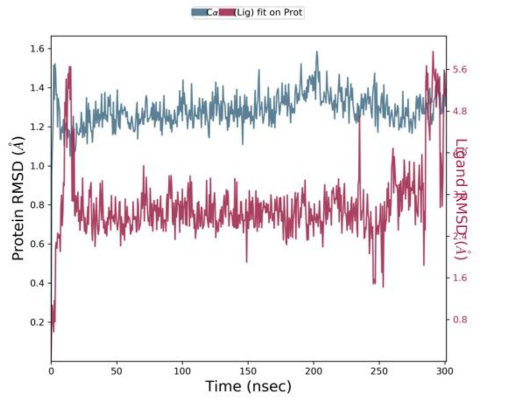

Thia, 9, AR

Figure S2. Protein and ligand RMSD profiles for compounds **6** and **9** during the 300 ns simulations. The large fluctuations on the ligand RMSD are due to the flipping of the distal biphenyl moiety, which in PTP-1B is solvent-exposed, and in AR is stabilized by two contiguous residues.

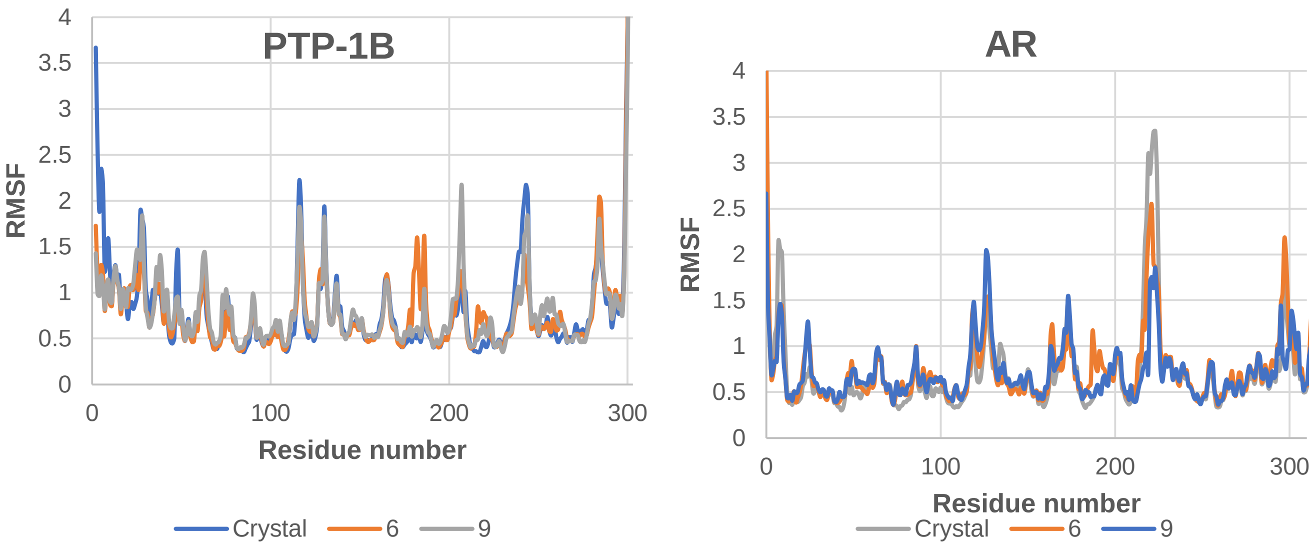

Figure S3. Root mean square fluctuations of the carbon alpha for the two targets during the simulations with the crystal inhibitor and compounds **6** and **9**.

Table S1. Ligand interactions in PTP-1B and AR. Score values under -6.0 kcal/mol were considered good values. Residues also involved in the interaction with the co-crystallized inhibitor are marked in red. The percentage of conformations in the largest cluster is reported in cluster size, while the most negative value is reported for the score.

	Score	Residue contacts for PTP-1B															Cluster size
		Y 46	R 47	N 48	V 49	N 181	F 182	C 215	S 216	A 217	G 218	I 219	G 220	R 221	Q 262	Q 266	
1	**-7.3**	■			■		■	■	■	■	■	■	■	■	■		38%
2	**-6.9**	■	■	■			■	■	■	■	■	■	■	■	■		41%
3	**-9.5**	■	■	■			■	■	■	■	■		■	■	■		66%
4	**-6.6**	■	■	■		■	■	■	■	■	■	■	■	■	■		46%
5	**-7.3**	■	■	■		■	■	■	■	■	■	■	■	■			60%
6	**-6.7**	■	■	■		■	■	■	■	■	■	■	■	■	■		48%
7	**-10.2**	■	■	■		■	■	■	■	■	■		■	■	■	■	28%
8	**-10.7**	■		■		■	■	■	■	■	■	■	■	■	■	■	29%
9	**-10.0**	■	■	■			■	■	■	■	■		■	■		■	45%

	Score	Residue contacts for AR																					Cluster size
		W 20	V 47	Y 48	Q 49	W 79	C 80	H 110	W 111	T 113	F 115	F 121	F 122	Y 209	W 219	C 298	A 299	L 300	C 303	Y 309	P 310	F 311	
1	**-11.6**	■		■		■		■	■	■			■		■	■		■		■	■	■	65%
2	**-10.5**	■	■		■				■	■	■		■			■	■	■	■			■	71%
3	**-9.0**	■	■			■			■	■		■	■			■		■	■				63%
4	**-11.9**	■		■		■		■	■	■	■		■	■	■	■		■		■	■	■	56%
5	**-10.1**	■	■	■		■	■	■	■	■	■		■		■	■		■	■	■	■	■	50%
6	**-6.8**	■	■	■		■		■	■				■			■	■	■			■	■	35%
7	**-11.1**	■	■	■		■		■	■	■	■		■			■		■	■	■	■		50%
8	**-9.7**	■	■	■	■	■	■	■	■	■	■		■			■	■	■	■	■			54%
9	**-6.4**	■	■	■		■			■	■	■		■		■	■	■	■	■				43%

Table S2. Calculated pharmacokinetic and genotoxic properties for compounds **1-9**

Cmpd	TPSA	Consensus Log P	Water solubility class	GI absorption	BBB Permeant	PgP substrate	Bioavailability score	PAINS alert	Ames toxicity	Carcinogenicity
**1**	46.53	3.87	Moderate	High	Yes	No	0.56	0	0.12	0.12
**2**	70.32	3.87	Moderate	High	Yes	No	0.56	0	0.04	0.04
**3**	46.53	4.07	Moderate	High	Yes	No	0.56	0	0.03	0.03
**4**	55.76	3.93	Moderate	High	Yes	No	0.56	0	0.67	0.67
**5**	79.55	4.15	Moderate	High	No	No	0.56	0	0.07	0.07
**6**	55.76	4.40	Moderate	High	Yes	No	0.56	0	0.04	0.04
**7**	89.93	3.81	Poor	High	No	No	0.55	0	0.58	0.58
**8**	113.72	4.07	Poor	High	No	No	0.55	0	0.54	0.44
**9**	89.93	4.28	Moderate	High	No	No	0.55	0	0.32	0.35
**PIO**	93.59	3.11	Moderate	High	No	No	0.55	0	0.30	0.30
**GLI**	121.98	3.58	Poor	Low	No	No	0.55	0	0.27	0.27

If the value is > 0.50, the compounds could be able to present the predicted activit[illegible]

Table S3. Acute toxicity profile predicted for compounds **1-9**, pioglitazone and glibenclamide

Compd	LD$_{50}$ (mg/kg)				Probability of inhibition/blockage (IC_{50} or Ki < 10μM)			
	Mouse		Rat		CYP450 isoform			hERG
	i.p.	p.o.	i.p.	p.o.	3A4	2D6	1A2	(Cardiotoxicity)
**1**	440	620	350	650	0.07	0.46	0.56	0.02
**2**	830	670	910	240	0.07	0.01	0.02	0.07
**3**	460	2400	580	500	0.08	0.03	0.03	0.05
**4**	540	1600	320	2400	0.09	0.04	0.12	0.04
**5**	630	920	1200	3700	0.09	0.01	0.01	0.14
**6**	300	1800	550	3400	0.09	0.04	0.02	0.10
**7**	330	870	140	33	0.28	0.05	0.53	0.76
**8**	500	630	330	190	0.32	0.02	0.04	0.88
**9**	280	800	200	400	0.36	0.06	0.09	0.47
**Pioglitazone**	440	1900	400	1100	0.22	0.03	0.08	0.21
**Glibenclamide**	980	1900	500	2400	0.20	0.08	0.01	0.60

REPRESENTATIVE SPECTRA OF FINAL COMPOUNDS 1-9

[4-(1-naphthylmethoxy)phenyl]acetic acid (1)

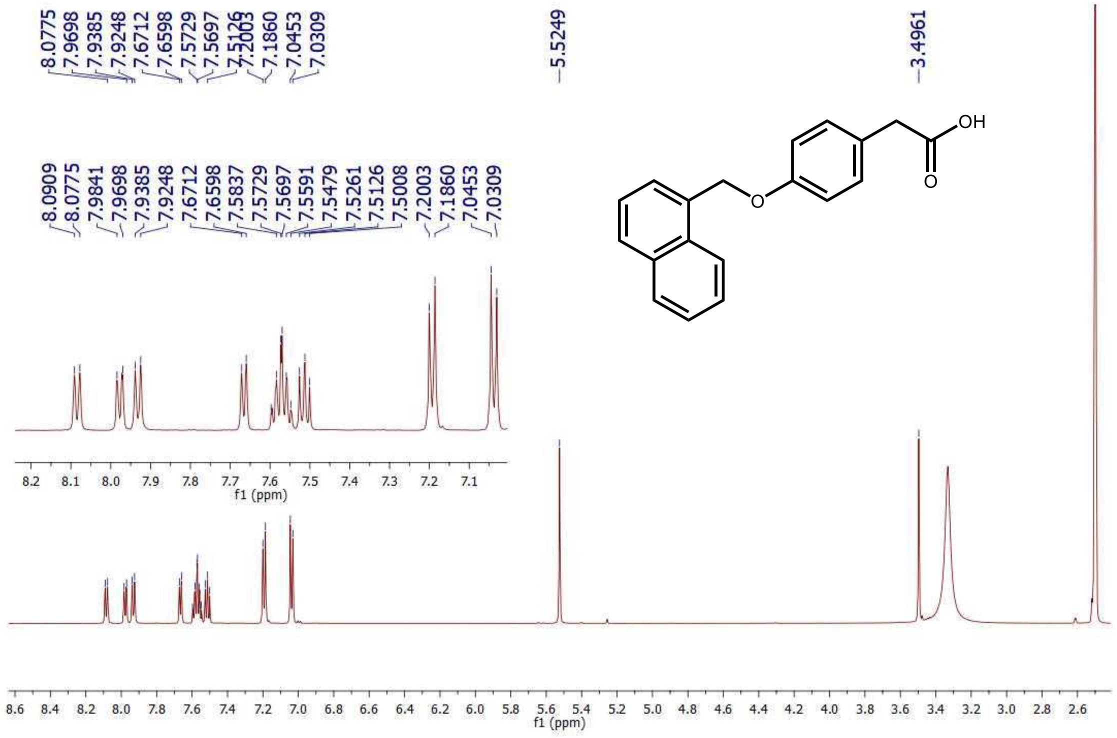

^{1}H NMR compound ***1***

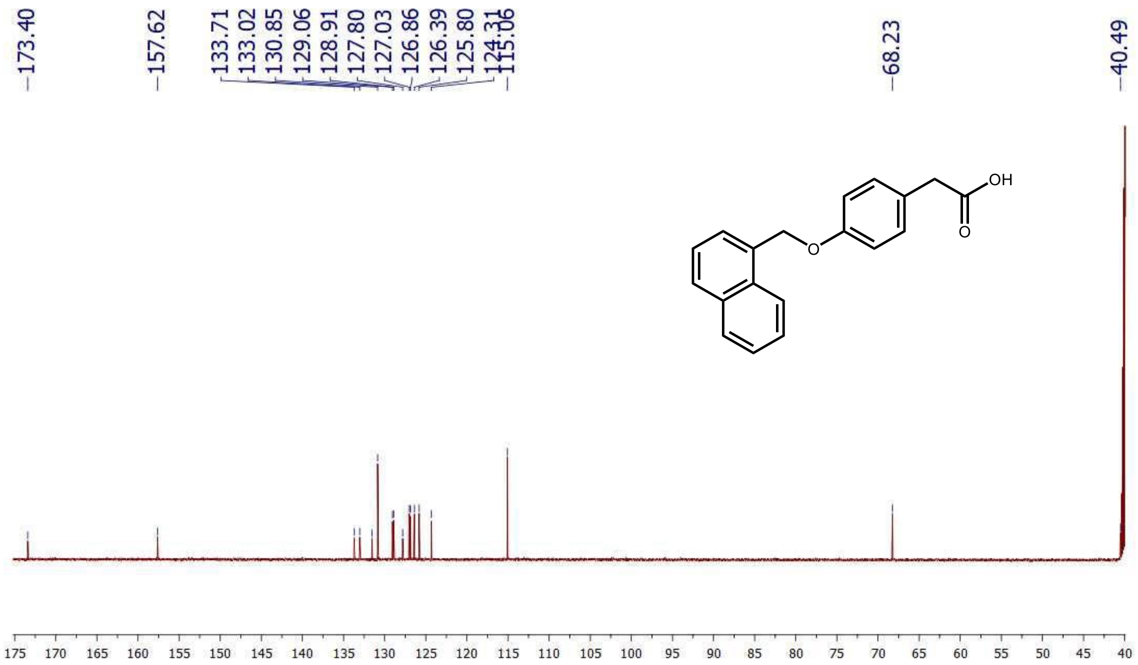

^{13}C NMR compound **1**

{4-[(2'-cyanobiphenyl-4-yl)methoxy]phenyl}acetic acid (2)

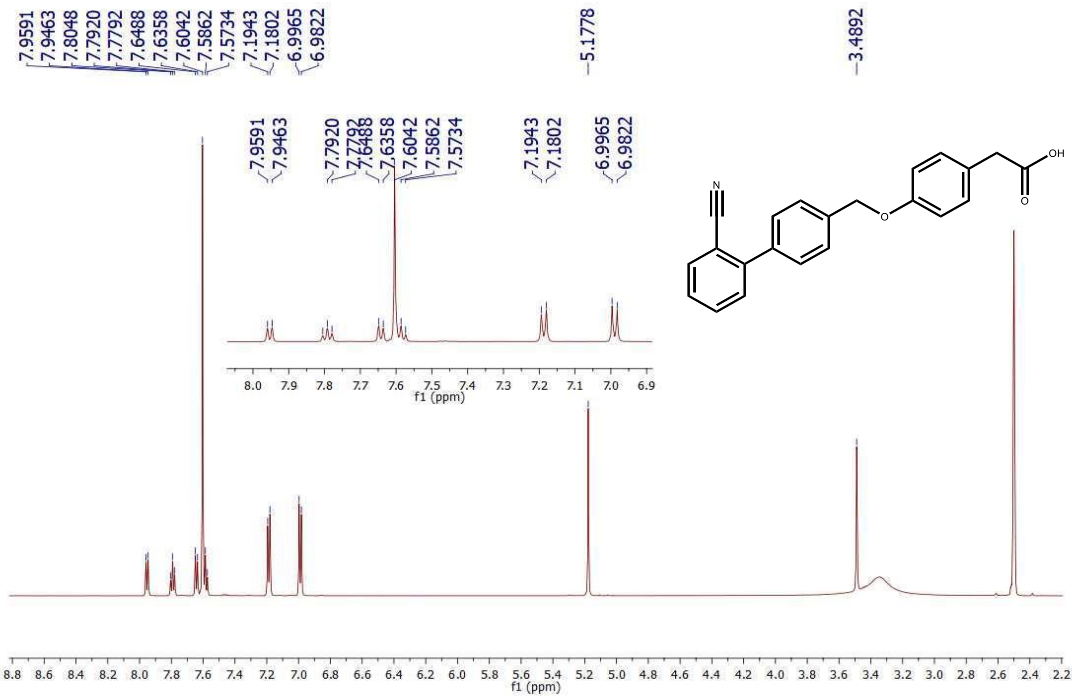

^{1}H NMR compound ***2***

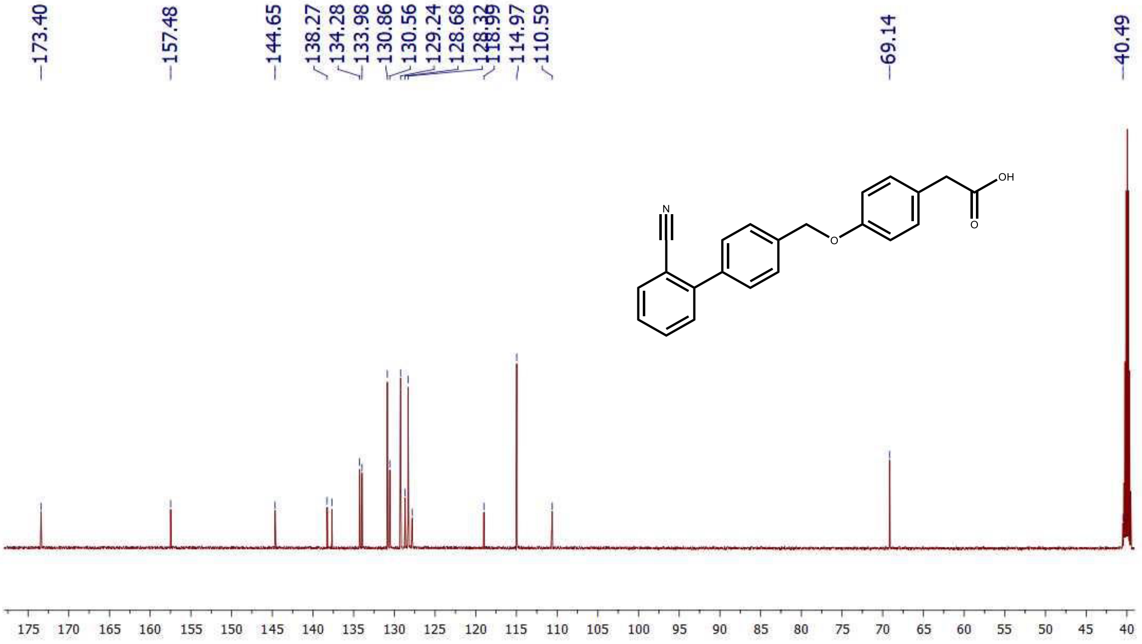

^{13}C NMR compound **2**

[4-(biphenyl-3-ylmethoxy)phenyl]acetic acid (3)

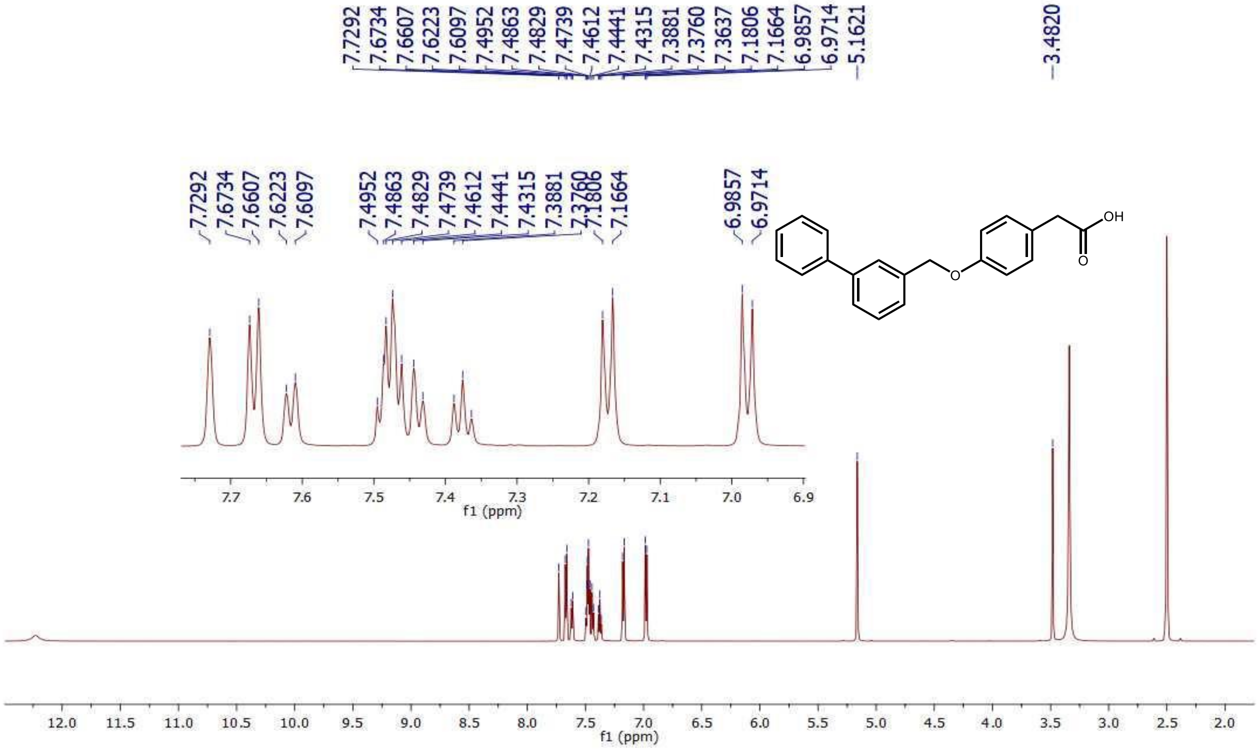

*^{1}H NMR compound **3***

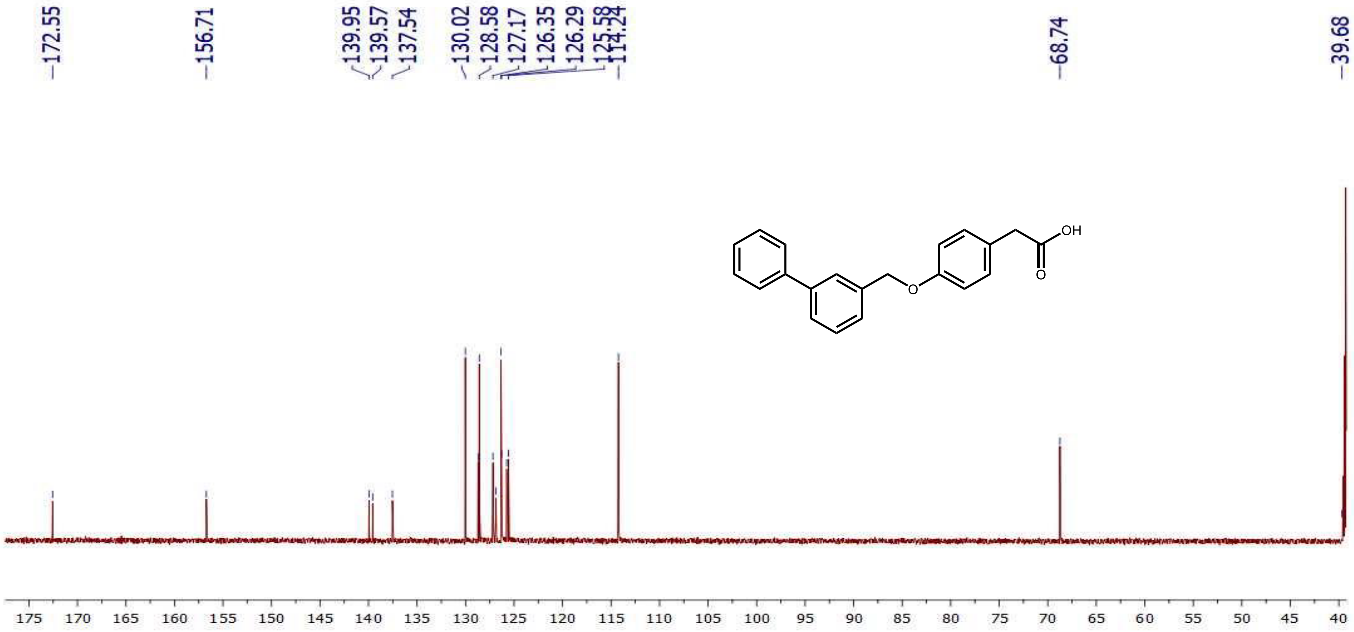

^{13}C NMR compound **3**

3-[3-methoxy-4-(1-naphthylmethoxy)phenyl]propanoic acid (4)

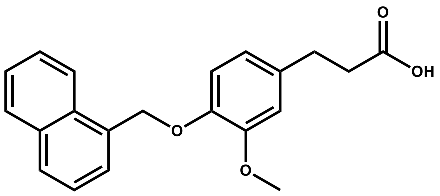

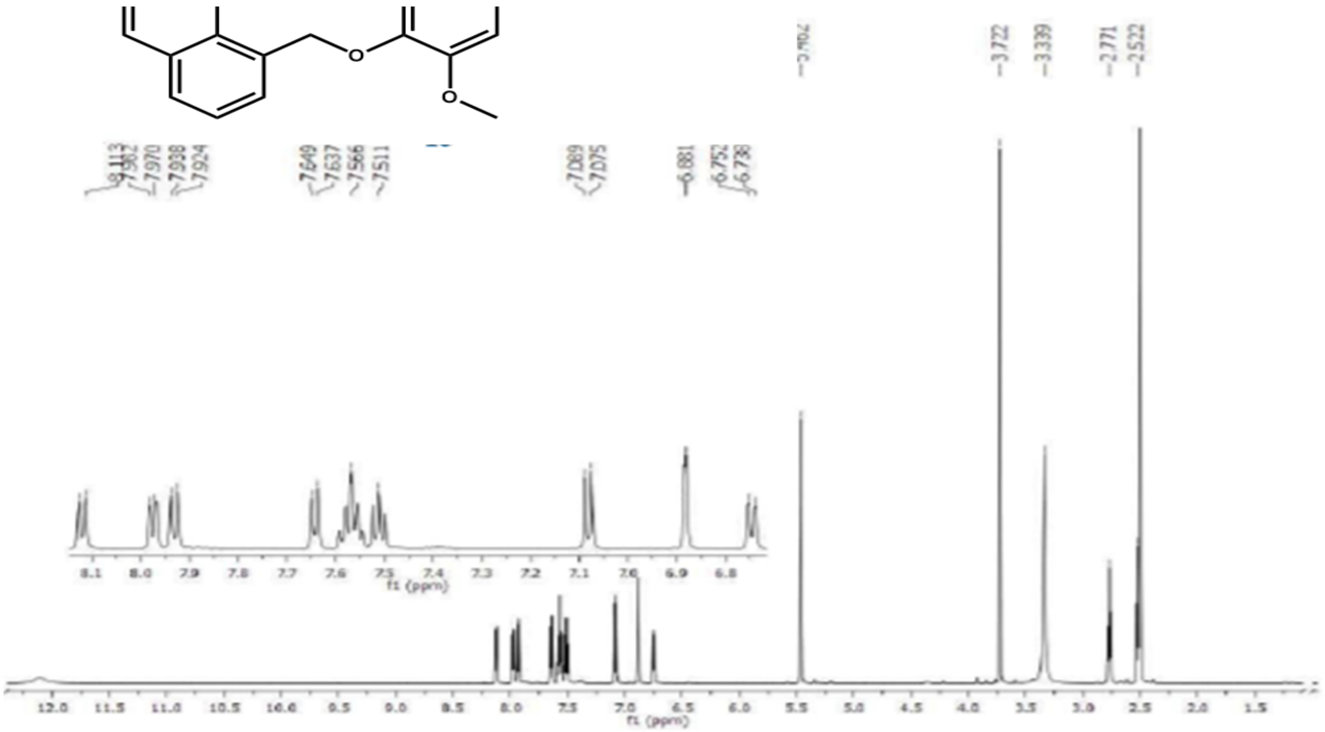

^{1}H NMR compound ***4***

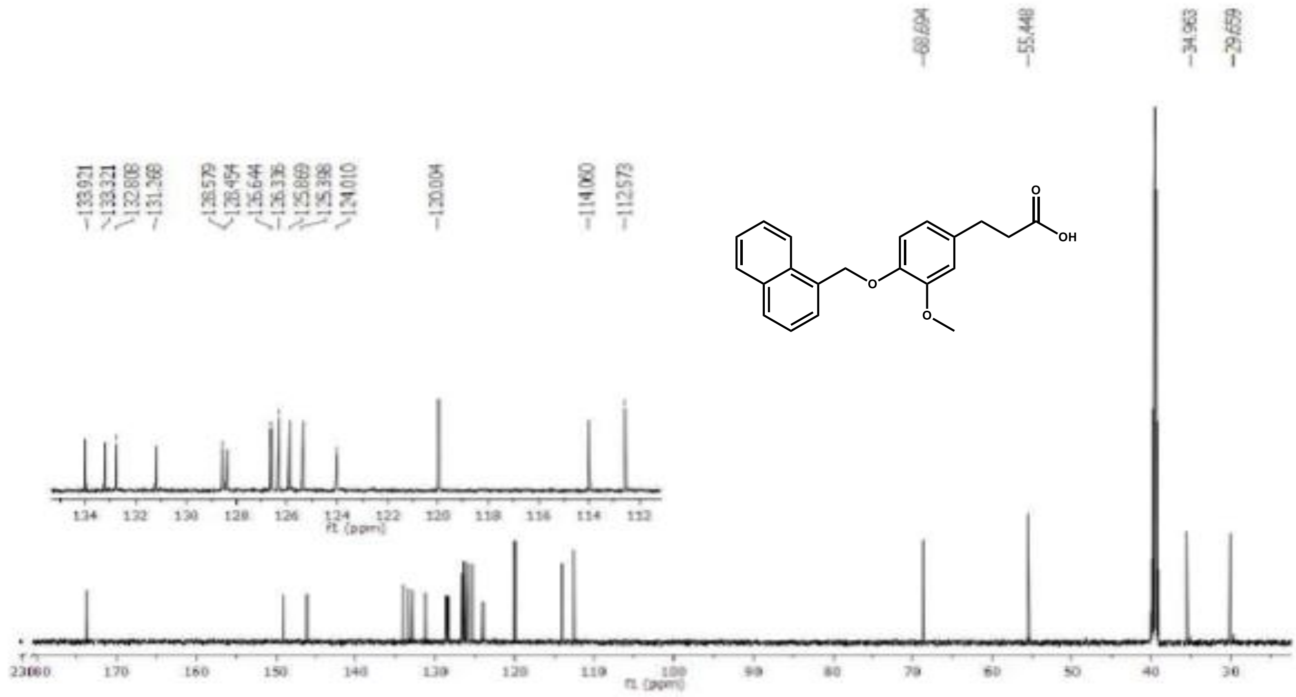

^{13}C NMR compound **4**

3-{4-[(2'-cyanobiphenyl-4-yl)methoxy]-3-methoxyphenyl}propanoic acid (5)

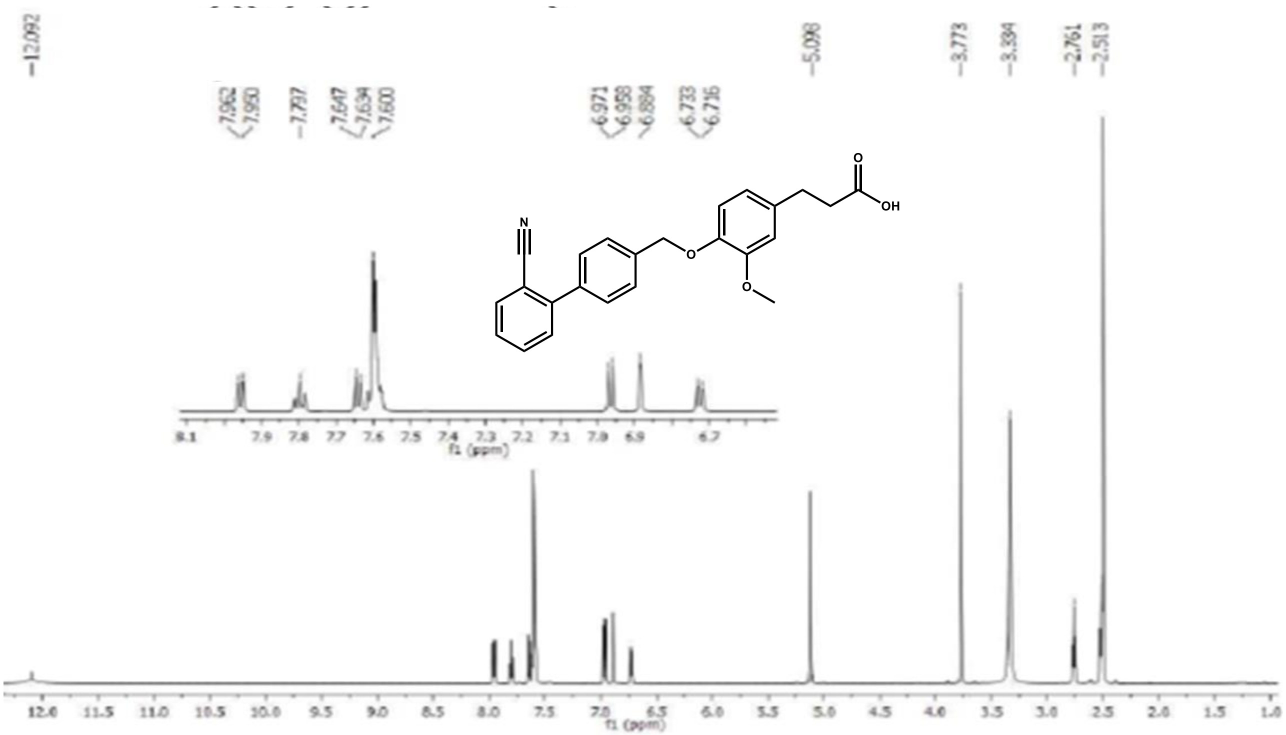

^{1}H NMR compound ***5***

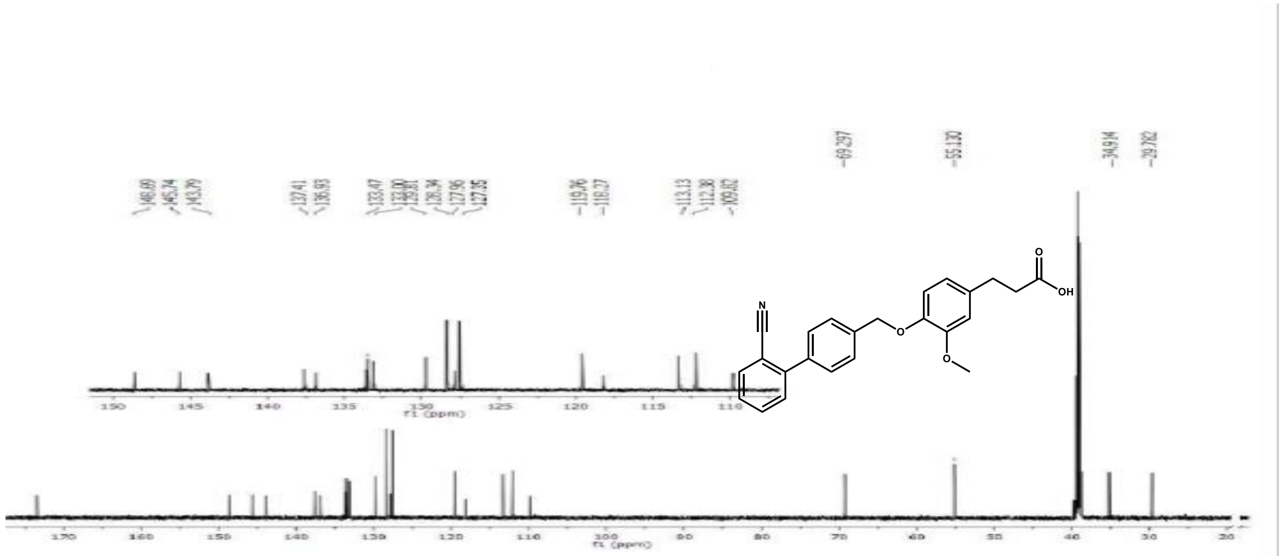

^{13}C NMR compound **5**

3-[4-(biphenyl-3-ylmethoxy)-3-methoxyphenyl]propanoic acid (6)

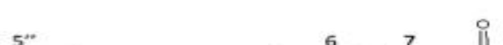

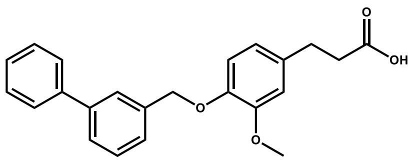

*^{1}H NMR compound **6***

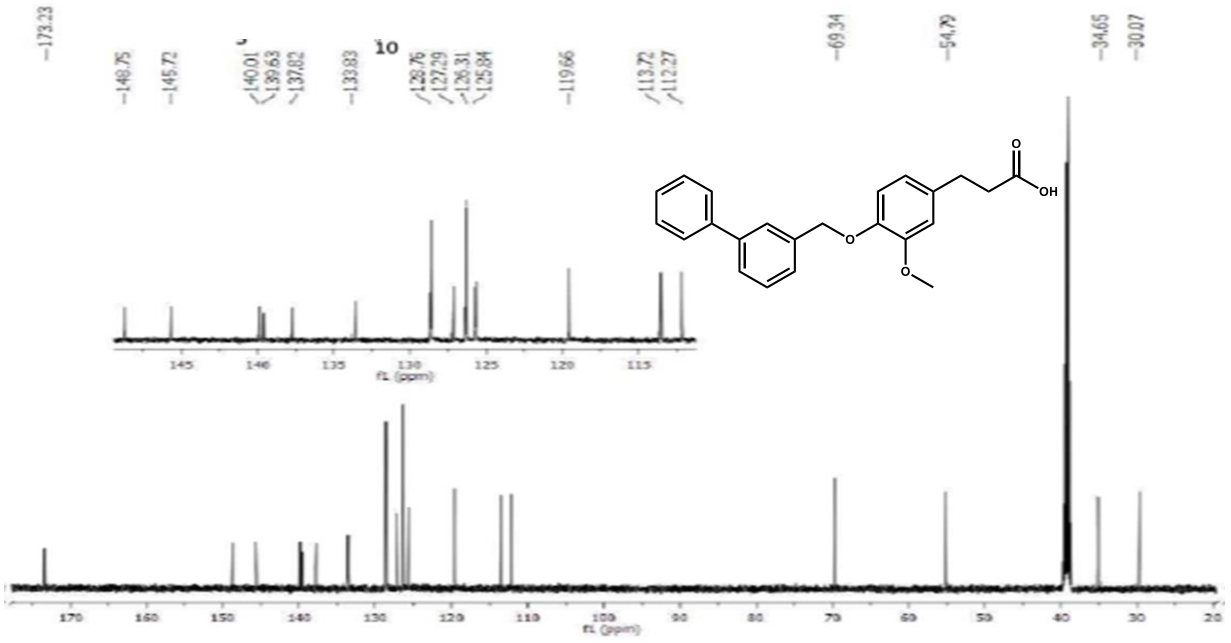

^{13}C NMR compound **6**

(5*Z*)-5-[4-methoxy-3-(1-naphthylmethoxy)benzylidene]-1,3-thiazolidine-2,4-dione (7)

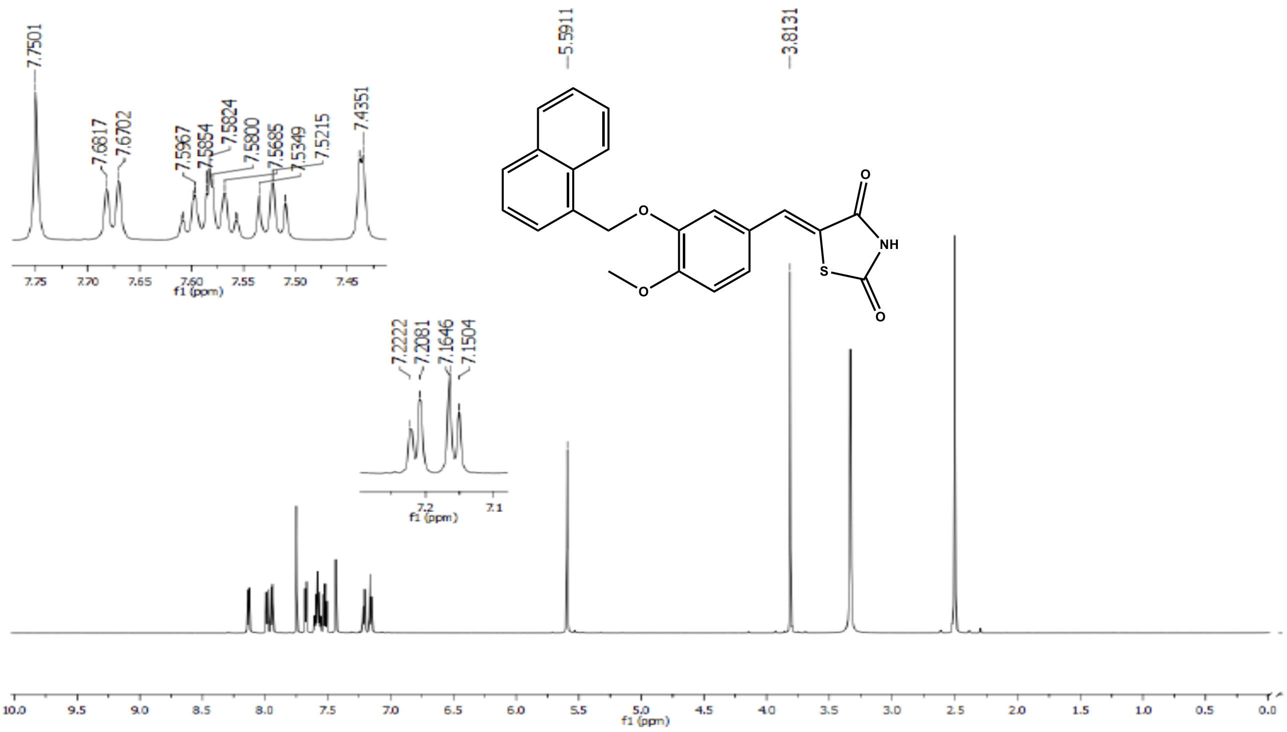

^{1}H NMR compound ***7***

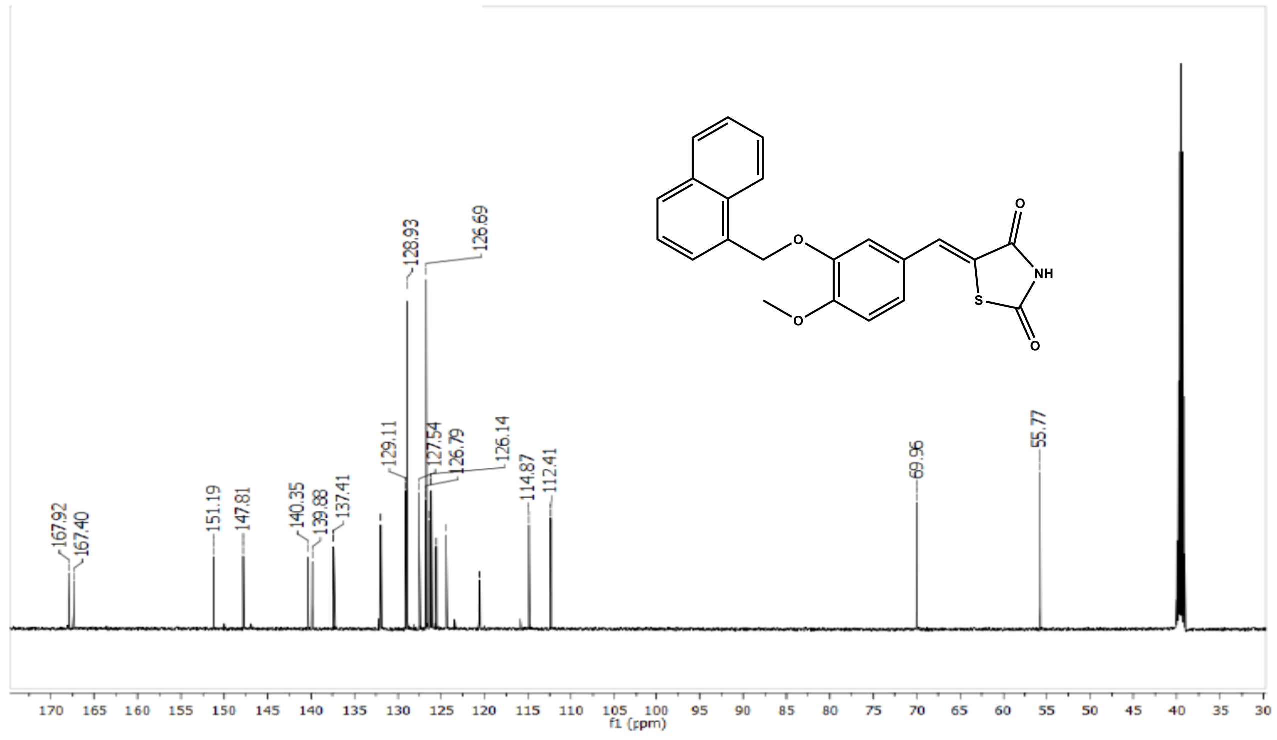

^{13}C NMR compound **7**

4'-({5-[(*Z*)-(2,4-dioxo-1,3-thiazolidin-5-ylidene)methyl]-2-methoxyphenoxy}methyl) biphenyl-2-carbonitrile (8)

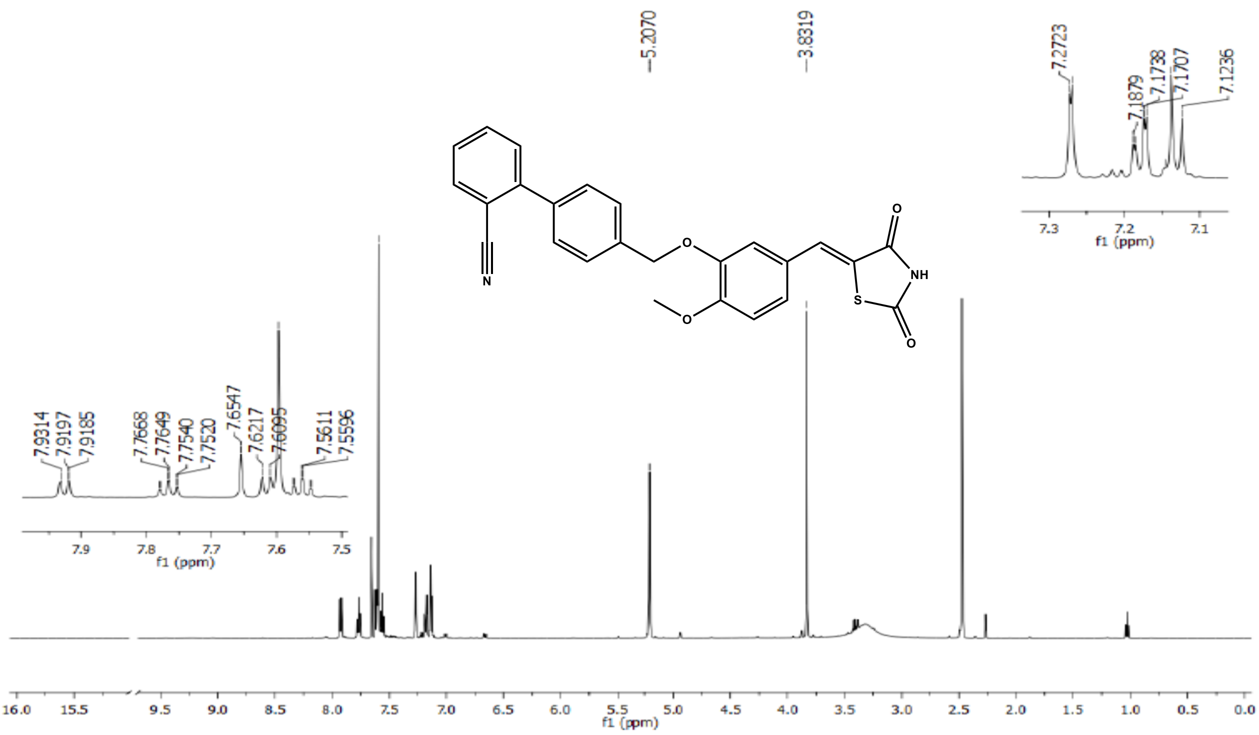

*^{1}H NMR compound **8***

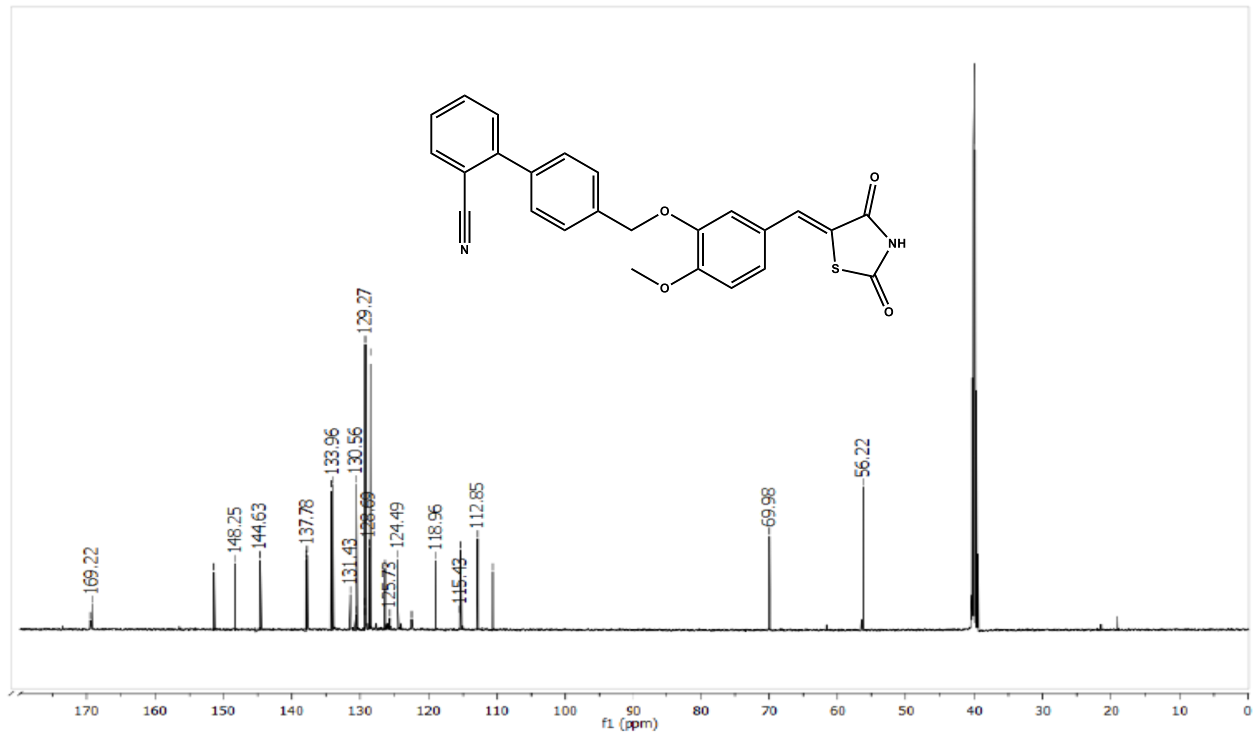

^{13}C NMR compound **8**

(5*Z*)-5-[3-(biphenyl-3-ylmethoxy)-4-methoxybenzylidene]-1,3-thiazolidine-2,4-dione (9)

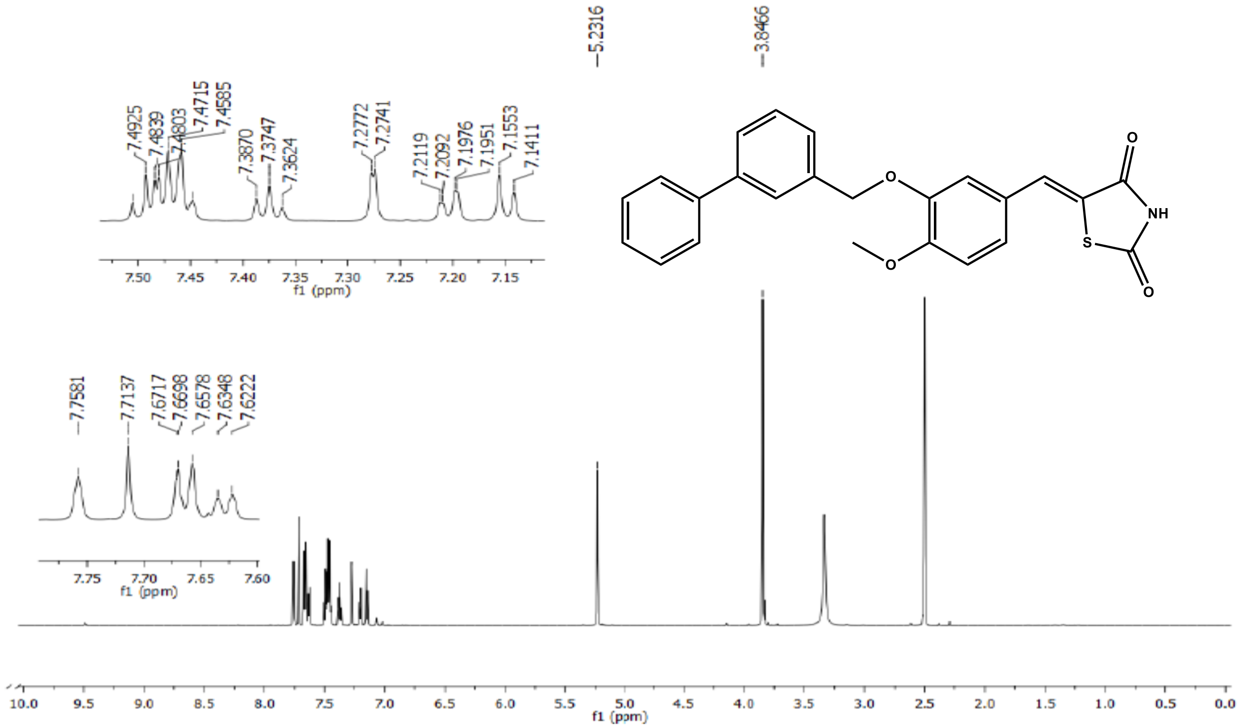

^{1}H NMR compound ***9***

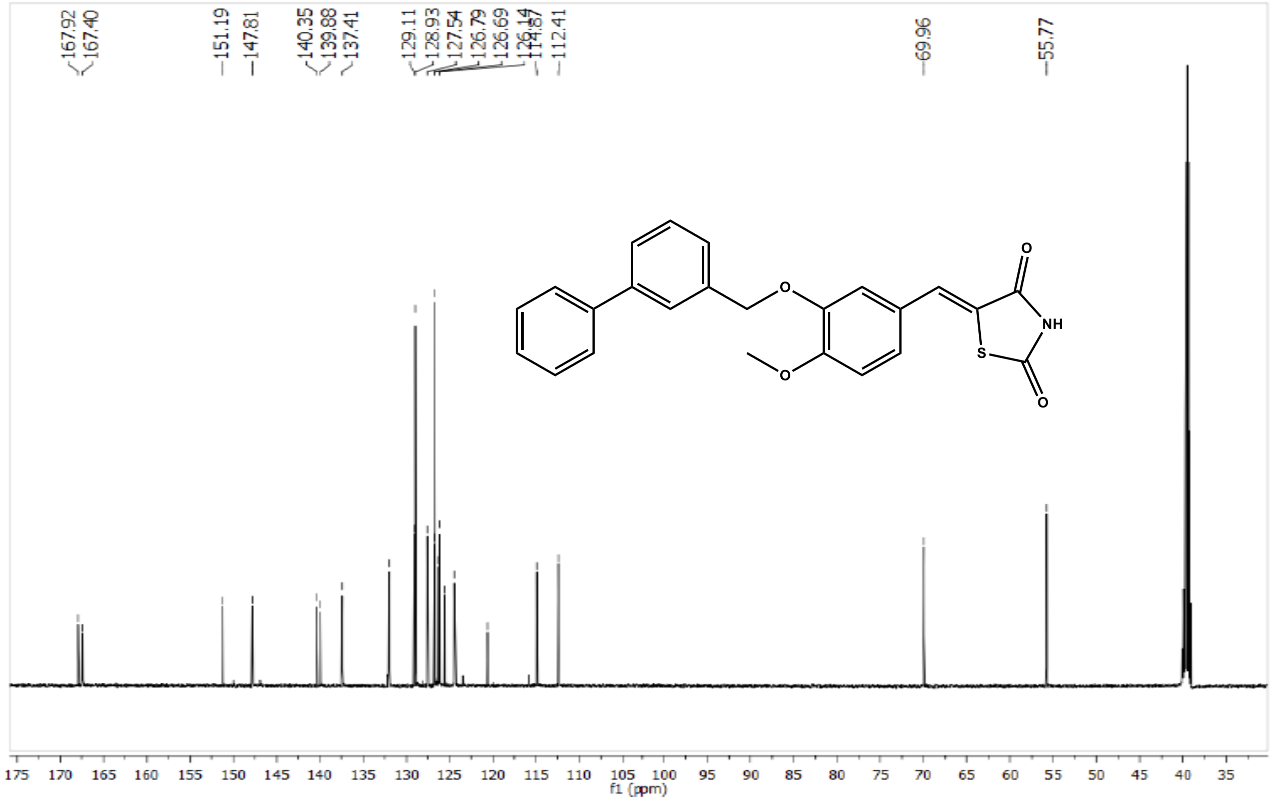

^{13}C NMR compound **9**
